# Recommendations to plan a national burden of disease study

**DOI:** 10.1186/s13690-021-00652-x

**Published:** 2021-07-07

**Authors:** Romana Haneef, Jürgen Schmidt, Anne Gallay, Brecht Devleesschauwer, Ian Grant, Alexander Rommel, Grant MA Wyper, Herman Van Oyen, Henk Hilderink, Thomas Ziese, John Newton

**Affiliations:** 1 Department of Non-Communicable Diseases and Injuries, Santé Publique France, 12 rue du Val d’Osne, 94415 Saint-Maurice Cedex, France; 2 Health Improvement, Public Health England, London, UK; 3 Department of Epidemiology and Public Health, Sciensano, Brussels, Belgium; 4 Department of Veterinary Public Health and Food Safety, Ghent University, Merelbeke, Belgium; 5 Public Health Scotland, Edinburgh, Scotland, UK; 6 Department of Epidemiology and Health Monitoring, Robert Koch-Institute, Berlin, Germany; 7 Centre for Public Health Forecasting, National Institute for Public Health and the Environment (RIVM), Bilthoven Utrecht, Utrecht, The Netherlands

**Keywords:** Burden of Disease, DALYs, YLL, YLD, *InfAct*, burden-eu, European Burden of Disease Network, Population health

## Abstract

**Background:**

The *InfAct* (Information for Action) project is a European Commission Joint Action on Health Information which has promoted the potential role of burden of disease (BoD) approaches to improve the current European Union-Health Information System (EU-HIS). It has done so by raising awareness of the concept, the methods used to calculate estimates and their potential implications and uses in policymaking. The BoD approach is a systematic and scientific effort to quantify and compare the magnitude of health loss due to different diseases, injuries, and risk factors with estimates produced by demographic characteristics and geographies for specific points in time. Not all countries have the resources to undertake such work, and may therefore start with a more restricted objective, e.g., a limited number of diseases, or the use of simple measures of population health such as disease prevalence or life expectancy. The main objective to develop these recommendations was to facilitate those countries planning to start a national burden of disease study.

**Results:**

These recommendations could be considered as minimum requirements for those countries planning to start a BoD study and includes following elements: (1) Define the objectives of a burden of disease study within the context of your country, (2) Identify, communicate and secure the benefits of performing national burden of disease studies, (3) Secure access to the minimum required data sources, (4) Ensure the minimum required capacity and capability is available to carry out burden of disease study, (5) Establish a clear governance structure for the burden of disease study and stakeholder engagement/involvement, (6) Choose the appropriate methodological approaches and (7) Knowledge translation. These were guided by the results from our survey performed to identify the needs of European countries for BoD studies, a narrative overview from four European countries (Belgium, Germany, The Netherlands and Scotland) and the summary of a comparative study of country health profiles with national health statistics.

**Conclusions:**

These recommendations as minimum requirements would facilitate efforts by those European countries who intend to perform national BoD studies.

**Supplementary Information:**

The online version contains supplementary material available at 10.1186/s13690-021-00652-x.

## Background

*InfAct* (Information for Action) is a European Commission Joint Action on Health Information with 40 partners across the EU (European Union) Member States aiming to develop a more sustainable EU health information system through improving the availability of comparable, robust and policy-relevant health status and health system performance data [1]. Through a series of three dedicated workshops, this joint action has emphasized the potential role of burden of disease (BoD) assessment and supported countries with an interest in developing a BoD study but who may lack the relevant expertise and capacity. The main aim of the *InfAct* BoD workshops was to promote the use of BoD, and to help countries integrate these methods into their routine public health activities and policy making and to improve the current European Union-Health Information System (EU-HIS). The BoD approach also offers opportunities for improvement in current practice, such as clearer and more concise documentation, and standardization of methodologies between countries, to make BoD assessments within Europe more comparable. BoD approaches in general provide comparative assessment frameworks, which include the key metrics such as years of life lost (YLL), years lived with disability (YLD), and disability-adjusted life years (DALYs) [2]. These estimates transform standard measures such as prevalence and mortality rates into aggregated or integrated measures, e.g. by applying severity distributions and disability weights (DWs) and thus produces new insights and adds value to standard population health assessment. Since BoD estimates combine measures of mortality and morbidity, they allow comparisons to be made between a broad range of conditions. This means that (bearing in mind the limits of the methods) the population health impact of conditions that primarily cause premature loss of life can be compared on a like-for-like basis with conditions that cause prolonged reductions in health. Estimates may also vary considerably between areas of a country, so subnational estimates are important to describe and highlight variations and inequalities. These types of estimates are key resources to use in knowledge exchange processes with the aim of developing proportionate prevention and interventions strategies to improve overall, and inequalities, in population health at national and subnational levels. The BoD approach has many applications and produces useful outputs, which make sense to policy makers even if they do not fully understand the methods used. However, the data requirements are substantial, and the methods used to produce the summary estimates are complex. Multiple assumptions, methodological choices, and often compromises, must be made to integrate information of different types from many sources. For those countries who do not want to undertake the task of preparing their own BoD estimates, it is possible to use readily available and recent national BoD estimates published by the Institute for Health Metrics and Evaluation (IHME). These are based on the well-resourced and long-standing Global Burden of Disease (GBD) study. However, data inputs into the GBD study do not always correspond to what stakeholders at national level consider the best available and most up-to-date information. The countries may also disagree with some of the assumptions made to generate BoD estimates. For these and other reasons, countries who have the resources and capability to do so have performed their own BoD studies [3–6].

For those countries who have limited resources, or no prior experience to undertake a BoD study, and intend to initiate a national BoD study in their country context, we propose some recommendations. These recommendations would support countries with a systematic approach for how to plan this study with restricted objectives (i.e. choose a limited number of diseases, use of simple measures of population health such as prevalence). To our knowledge, no such recommendations are available to guide countries to initiate, or plan, a national BoD study. The main objective to develop these recommendations was to facilitate those countries planning to start a national burden of disease study.

## Burden-eu Network

In 2019, the burden-eu (European Burden of Disease Network) COST (European Cooperation in Science and Technology) Action (CA18218) was launched, with an aim to develop a technical platform to integrate and strengthen capacity in BoD assessment across Europe and beyond [7]. The burden-eu COST Action has four priorities for intensified collaborations: (i) increased interaction between existing BoD efforts; (ii) technical capacity building at country level; (iii) a platform to support methodological advances; and, (iv) an actionable understanding of the process underlying knowledge translation [8]. Aforementioned, the *InfAct* project has emphasized the potential role of BoD assessment; supported countries interested in developing a BoD study and improved specific expertise to develop their capacity. This technical platform would support to achieve these objectives and would continue to establish and strengthen the scientific collaborations to integrate BoD approaches in routing public health activities.

## Methods

To develop the recommendations to plan a national burden of disease study, we carried out three main activities under *InfAct* project: (1) a survey was performed to identify the needs of European countries to perform a national burden of disease (BoD) study (additional file 1), (2) the *InfAct* project partners who are performing their own BoD studies (i.e., Belgium, Germany, The Netherlands and Scotland), were asked to provide an overview of key elements such as data sources used, methodological approaches applied, methodological challenges and related solutions, implication of BoD estimates in health policy and perspectives (additional file 2) as a narrative overview, and (3) using the ‘standard’ GBD metrics available in the GBD 2017 study, we have extracted a series of country health profiles of European countries from IHME website (https://vizhub.healthdata.org/gbd-compare/ and http://ghdx.healthdata.org/gbd-results-tool). We asked the *InfAct* partners to compare these estimates with their national health statistics (additional file 3).

Based on the results of these studies and the inputs from the experts of this domain, we developed these recommendations.

## Results

### Outputs of burden of disease activities performed under InfAct

We performed a survey in May 2019 among European countries to identify the current needs to perform BoD studies (Additional file 1). Among 25 participating countries, 72 % mentioned that they have not carried out any BoD study in the past and have no experience from which to develop a case study on BoD. A few have already performed national BoD studies (i.e. Belgium, Germany, The Netherlands and Scotland). These countries were asked to provide a narrative overview of their experience of performing BoD studies (Additional file 2) [9]. These countries have calculated, or are calculating, BoD estimates not only at the national level but also at subnational levels. Their experience could support and guide others to initiate and integrate the burden of disease approaches into their routine public health activities. Then, we performed a study comparing the country health profiles (i.e., providing a measure of priority health conditions and risk factors, a summary breakdown of major causes, and an appreciation of health sector performance) from the GBD study with national health statistics [10] (Additional file 3). Many important differences were highlighted because countries were using different data sources compared with the GBD study, and different methods (such as differences in the standard population used in age-standardized rate calculations).

### Recommendations

In collaboration with experts from *InfAct* project and burden-eu network, we have developed some recommendations for countries who are planning to develop national BoD studies. These recommendations could be considered as minimum requirements for those countries planning to start a BoD study. These were guided by the results from our survey performed to identify the needs of European countries for BoD studies, a narrative overview from four European countries (Belgium, Germany, The Netherlands and Scotland) [9] and the summary of a comparative study of country health profiles with national health statistics [10]. Wide adoption of these recommendations could help harmonise and facilitate efforts by those European countries who intend to perform their national BoD studies.

#### Define the objectives of a burden of disease study within the context of your country

The BoD study is “a systematic, scientific effort to quantify the comparative magnitude of health loss due to diseases, injuries, and risk factors by age, sex, and geographies for specific points in time”, or, “a comparative assessment framework, which includes the key metrics of years of life lost (YLL), years lived with disability (YLD), and disability-adjusted life years (DALYs)” [2]. However, European countries need to define the objectives of the BoD study within the context of their country. While the standard definition of a BoD study is based on the use of DALYs for quantifying the population health impact of all relevant diseases and risk factors, we acknowledge that not all countries have the resources to achieve this comprehensive assessment, and may therefore embark with a more restricted scope, e.g. study a limited number of diseases, or the use of simple measures of population health such as disease prevalence or life expectancy (LE).

#### Identify, communicate and secure the benefits of performing national burden of disease studies

Policymakers need to be informed about the relative scale of different health problems in the population, the groups that are particularly at risk, and the trends in the state of health over time. In addition, a representative estimate of the population’s health status can be used for determining the expected health care use and is vital evidence to use when prioritizing effective interventions and evaluating their impact and cost-effectiveness [11].

The following is a list of some potential uses of BoD estimates:

- ○ Health and policy improvement
  - ▪ Drawing attention to the effects of non-health outcomes on overall population health and rank the impact of diseases on population health expressed in terms of lost life years due to illness (YLD) and death (YLL) in a single summary measure (DALY).
  - ▪ Comparing the health of one national or subnational population with that of another (including international benchmarking).
  - ▪ Monitoring changes over time in the health of a given population.
  - ▪ Identifying and quantifying health inequalities within countries.
  - ▪ Priority setting by health condition and risk factor.
  - ▪ Informing debates on priorities for health service delivery and planning.
  - ▪ Informing debates on priorities for research and development.
- ○ Resource allocation
  - ▪ Rational and proportionate allocation of resources: trends in specific conditions and differences in outcomes across ages and between sexes can yield insights about where new investments in health resources are needed.
  - ▪ Analysing the potential benefits of health interventions for use in cost-effectiveness analyses [12]..
- ○ Data improvement and quality of the Health Information System
  - ▪ Performing a national BoD study helps to appraise and improve the completeness and quality of available data to be used, consequently this helps to improve the country’s health information system.
- ○ Helping to build capacity
  - ▪ Performing a BoD study helps to build capacity in BoD assessment, and population health and epidemiology in general.
  - ▪ Relevant training programmes and workshops can increase awareness and build local capacity and expertise to use BoD methods.
- ○ Strengthening collaborations
  - ▪ BoD work can be used to strengthen collaborations within a country and with other countries and international organisations (such as WHO, IHME and through the burden-eu COST Action) to integrate and strengthen capacity in BoD assessments across Europe and beyond [8].

#### Secure access to the minimum required data sources

As a minimum data requirement to perform a BoD study, high quality cause specific mortality data (best differentiated at the level of three- or four-digit ICD-10 codes [International statistical Classification of Diseases and Related Health Problems-10th Revision]) and other disease-specific statistics are needed. These may include one or more of the following sources: national health administrative data sources (general practitioner registration, hospital discharge data and/or health insurance data), available disease-specific registries, health surveys, vital and causes of death statistics (i.e. census, birth, and death registries). Additional information can be integrated from the scientific literature or from the GBD study. Data used could be either linked or unlinked, at the individual level or at an aggregated level. It is essential to use the best available high-quality data. Which data sources are needed also depends on the objectives of the analyses. As a first step, causes of death and vital statistics can be used to calculate YLL, and later national health administrative data sources, registries, or health surveys can be used to determine disease prevalence and YLD.

#### Ensure the minimum required capacity and capability is available to carry out burden of disease study

BoD assessments are a collaborative effort, particularly when they are performed across a widespread range of health conditions and risk factors. It provides an opportunity to mobilise the scientific knowledge and competencies with a multidisciplinary approach and networking from various domains. To perform a BoD study, the minimum required workforce would include epidemiologists, data managers, biostatisticians/statisticians, public health experts and demographers.

#### Establish a clear governance structure for the burden of disease study and stakeholder engagement/involvement

National public health institutes are ideally placed to be responsible for or for co-ordinating, national BoD studies in collaborations with various partners to share their expertise. Stakeholder engagement is important at every stage [13] to share information, coordinate and obtain experts opinion on BoD indicators.

#### Choose the appropriate methodological approaches

Countries undertaking their own analyses need to select appropriate approaches that fit within their country contexts. When making methodological choices about BoD methods, one should be aware of certain standardized methods proposed by the IHME if comparability with GBD is an aim. If country and subnational contexts are different from the GBD study, then these estimates will retain strong within-country value but their utility in comparison with GBD estimates are limited. This may not matter depending on the purpose of the estimates. Some reference guides are available that can help countries to follow various methodological approaches. For example, WHO 2001 a practical guide on national BoD studies [14].

A recent study highlighting the key methodological decisions in national BoD assessment [15], the narrative overview of national BoD studies [9] and the comparison of country health profiles with national health statistics [10] have emphasized the importance of the following aspects of the methods used:

- ○ Methodological choices comprise (some of these are optional ones, others demand a choice):
  - ▪ Use of national or GBD (standard/ideal) life tables to estimate YLL [16]: It is important to take into account that choosing a local life table for each year of estimation may invalidate comparisons over time unless the life table is retrospectively applied to previous years.
  - ▪ Methods used to redistribute garbage codes/ill-defined death codes and invalid ICD-10 codes when making counts of death by cause [4, 17]: The choice of an appropriate method to redistribute the garbage codes/ill-defined deaths codes is based on the types of identified garbage codes/ill-defined death codes in the given mortality database.
  - ▪ Use of bespoke national or GBD disability weights (DWs) and severity distributions when calculating YLDs [18]: The GBD DWs derive weights for all conditions in a consistent manner. Choosing an alternative set of DWs does invalidate comparison with countries the used a different set of DWs.
  - ▪ Multimorbidity adjustment method [19].
  - ▪ Methods for estimating uncertainty levels is optional whether to quantify uncertainty, and if so, whether to use a quantitative or qualitative approach.
  - ▪ Distribution of potential risk factors in population by age, sex and geographical level to calculate the relative risk (RR), attributable fraction (AR) and population attributable fraction (PAF) and to calculate risk attribution to disease burden.
- ○ Geographic level (i.e., municipalities, metropolitan, subnational and national level).
- ○ Choice of a reference population used in age-standardized rates calculations: The choice of a standard population is an arbitrary one. In countries with large population differentials between population groups, the choice can have a considerable impact on results. For European countries, it is likely to be most appropriate to use the 2013 European standard population (ESP2013) [20]. For within country comparisons a local standard population may be more appropriate especially if the country has a markedly different age/sex structure from the rest of Europe.
- ○ Data standards should align with existing data and metadata standards where appropriate: i.e. WHO approved terminologies/ontologies including ICD-10.
- ○ BoD indicators should be reported according to the GATHER (Transparency, as per Guidelines for Accurate and Transparent Health Estimates Reporting) guidelines [21].

#### Knowledge translation

The communication of BoD estimates to the policymakers and other public stakeholders is key to evidence-informed policymaking and to support decision making about the allocation of resources. The results can be communicated by, for example, policy briefs, infographics, flow charts, powerful graphics, use understandable language. These ways of communication can be seen in a larger context of knowledge translation [22]. Applying the concept of knowledge translation can also be helpful to highlight research and data gaps or areas that needed to be improved.

## Discussion

The importance of BoD assessment has been well acknowledged. The BoD assessments could be performed as a national BoD study, or through collaborating with IHME’s GBD study or using GBD estimates of prevalence or risk factors for some diseases where the data is not available in the country assessments for national BoD study. These approaches have their advantages and disadvantages. However, countries can make a decision to choose one approach for BoD assessment based on their country context.

### National burden of disease approach

#### Advantages

There are several advantages of performing a national burden of disease study: *First*, it strengthens the scientific collaborations within a country and across countries through the involvement of disease, risk and methodology experts. *Second*, it helps developing expertise in BoD studies including design, data preparation, analysis, interpretation and translation of results to policymakers. *Third*, access to local data sources that are not publically available to researchers outside the country, could be used to produce local estimates. *Fourth*, using the best available and updated data can help to judge the quality of local data sources. Furthermore, it helps to identify the data gaps and to improve the quality of data. *Fifth*, it helps to establish a close relationship/link to national and subnational stakeholders and policymakers and to communicate the results for rational and proportionate allocation of resources. *Six*, it strengthens the transparency and accuracy in estimates based on well-known data sources and makes it easy to understand the methodological approaches used. *Seventh*, it allows sub-national comparisons and can take into account the differences in the local health care system and surveillance.

#### Disadvantages

Despite several advantages, there are some downsides of the national burden of disease studies: *First*, it is a huge scientific and collaborative effort that requires resources and capacities to manage that. If countries have limited resources, it could be challenging. *Second*, lack of skills and experience in certain methodological aspects such as redistribution of garbage codes for causes of death, dealing with the uncertainty of estimates and correction of measurement bias of prevalence/incidence data. *Third*, national BoD approaches always take a lot of decisions making and make it difficult to compare the results directly to other studies. Greater transparency and the possibility of sub-national comparisons are bought at the cost of an overwhelming loss of international comparability. *Fourth*, in national BoD assessments where either more than one data source is available or a single data source, is worthy and better to use than relying on model estimations that can be based on no data from the country. However, the use of a single data source to reflect the disease occurrence may pose the risk of compositional bias in the estimates.

### Collaborating with IHME’s GBD approach

#### Advantages

 There are several advantages in collaborating with the IHME’GBD approach. *First*, the estimates produced by the GBD approach are comparable across countries. *Second*, GBD applies specific methods to address the uncertainty bounds reflecting uncertainty in data inputs, data manipulations to correct for biases, and model selection. *Third*, the GBD approach evaluates all appropriate data sources to avoid the compositional risk of bias due to a single data source. *Fourth*, GBD produces a time series of estimates over the past 3 decades. *Fifth*, countries with limited resources can share their data with IHME (respecting GDPR), this collaboration can improve the quality of data sources used by IHME and can benefit the country as well. *Sixth*, there is a possibility of working together based on a MoU (Memorandum of Understanding) allowing some forms of technical exchange for methods while following your approach. *Seventh*, GBD has been fully compliant with GATHER guidelines since these were established and produces a wealth of information describing methods, data sources and changes in modelling decisions.

#### Disadvantages

Despite some advantages, there are some downsides of this approach: *First*, GBD assessment takes into account different data sources and the local data sources may not be accessible that are used in the country to calculate the basic epidemiological indicators (prevalences/incidences) or the recent estimates may not be communicated with the IHME. Therefore, the calculated estimates may not reflect the updated country situation. *Second*, the GBD approach involves complex modelling and computational procedures that make it challenging to understand the process behind these estimations. *Third*, there are some shifts in results from one GBD version to another and sometimes even for the same year and some of GBD’s estimates fluctuate over time reflecting new data or new methods. However, data sparsity and biases affecting data sources can be challenging to make estimates more stable. This influences the knowledge translation of estimates with national stakeholders, so when results deviate that makes it difficult to communicate the underlying reasons of differences due to the complexity of the broad modelling process. As national public health agencies are accountable to Governments and therefore, are expected to provide the relevant explanation.

### Current focus and remaining challenges

The current focus of these recommendations is to provide an opportunity to mobilise existing scientific knowledge and competencies with a multidisciplinary approach and to encourage networking from various domains at European and international levels. The proposed recommendations could be considered as minimum requirements for those countries planning to start a BoD study. To implement and to integrate BoD approaches in steering public health activities, the technical platform of burden-eu COST Action would provide operational support to achieve these objectives and would continue to strengthen scientific collaborations.

The previous study on comparisons of country health profiles with national health statistics has highlighted the importance of key aspects such as differences in data sources, choice of a standard population in age-standardized rate calculations, and the differences between methods used to calculate BoD estimates when developing BoD studies [10]. One of the key challenges in producing the burden of disease indicators is the availability of updated, high quality, complete and reliable data sources. These aspects can have a substantial impact on the certainty upon which we can place upon estimates. These are the key areas where countries could invest to improve the quality of available data sources for reliable estimates and to build their capacity and skills for calculating BoD estimates at a small scale (i.e., including minimum number of diseases).

The strength of this study is that these recommendations were reviewed by BoD experts and may facilitate efforts to start, or to intergrate, the BoD approach in routine public health activities. These recommendations were developed based mainly on the current experience of using BoD approaches in the national public health insititutes of European Union countries and did not take into account the full experience of those research insitutes in each country who may be involved in additional BoD activities.

## Conclusions

The *InfAct* project is a European Commission Joint Action on Health Information, which has promoted the potential role of the burden of disease (BoD) approaches to improve the current European-Health Information System (EU-HIS). The *InfAct* BoD workshops have raised the awareness of the concept, the methods used to calculate estimates and their implications and uses in policymaking. The BoD is a collaborative effort built on shared experiences and techniques. The BoD approach is a systematic and scientific effort to quantify and compare the magnitude of health loss due to different diseases, injuries, and risk factors with estimates produced by demographic characteristics and geographies for either a single geographical location or several locations of interest for specific points in time. We acknowledge that not all countries have the resources to undertake such work, and may therefore start with a more restricted scope, e.g., a limited number of diseases, or the use of simple measure of population health such as disease prevalence or life expectancy. For those countries planning to start a BoD study, these recommendations and adoption of these proposed minimum requirements, would promote to harmonise and facilitate efforts by European countries.

## Supplementary Information

**Additional file 1.** It describes the summary of survey results and the format of questionnaire used to identify the need of European countries for BoD studies.

**Additional file 2.** It describes the experience of countries performing their own burden of disease study as a case study report.

**Additional file 3.** It describes the study results comparing the country health profiles and national health statistics and inputs from different countries.

## Data Availability

Not applicable.

## Abbreviations

InfAct: Information for Action (i.e., joint action of European countries to establish a more sustainable health information system)
burden-eu: European Burden of Disease Network
COST: European Cooperation in Science and Technology
BoD: Burden of Disease
EU: European Union
EU-HIS: European Health Information System
IHME: Institute for Health Mertics and Evaluation
GBD: Global Burden of Disease
DALYs: Disability Adjusted Life Years
DWs: Disability Weights
YLL: Years of Life Lost due to premature mortality
YLD: Years Lived with Disability
ESP2013: European Standard Population 2013
ICD: International Statistical Classification of Diseases and Related Health Problems
WHO: World Health Organization
GATHER: Guidelines for Accurate and Transparent Health Estimates Reporting
MoU: Memorandum of Understanding
