# Supplementary material for: Recommendations to plan a national burden of disease study
Source: Arch Public Health. 2021 Jul 7;79:126. doi: 10.1186/s13690-021-00652-x (PMC8262070; doi:10.1186/s13690-021-00652-x)
Supplement: Supplementary file 1 — Additional file 1. It describes the summary of survey results and the format of questionnaire used to identify the need of European countries for BoD studies. [file 13690_2021_652_MOESM1_ESM.pdf]

**Additional file 1: Summary of Survey results**

We performed a survey among European countries to identify the current needs for BoD studies to integrate into routine public health activities in May 2019. This survey addressed following aspects: BoD support required (i.e., for methodology, practical exercises with test data, interpretation of BoD data, and translation of BoD indicators into health policy), availability and accessibility of different data sources, existing workforce and governance of BoD studies (see annex 1.1).

The survey invitation was sent to 27 EU/EEA countries and the response rate was 93% (25/27).

Among 25 countries, 72% mentioned that they have not carried out any BoD study in the past and have no experience to develop a case study for BoD. These countries mentioned that they are required the support for BoD methodology (i.e., 19 countries), practical exercises to estimate the BoD indicators (i.e., 17 countries), interpretation of BoD data (i.e., 19 countries) and translation of BoD data into policy (i.e., 14 countries). Data from different sources (i.e., electronic health records, disease registries, health surveys and vital statistics) are available in all responded countries. Among 25 countries, almost 80% have access to the data at national level, 72% at subnational level and only 20% can access at metropolitan level. The majority of the countries have necessary workforce to conduct a BoD study including public health experts, epidemiologists and statisticians. Very few have the health economists and demographers. For the governance of BoD studies, 80% of responded countries mentioned that public health institutes should have the governance to perform the BoD studies.

24 **Annex 1.1: Survey questionnaire**

| **A. Background information** | Responses |
| --- | --- |
| Name | |
| Country | |
| Institution | |
| Responsible for: | |
| Have you carried out any previous BoD work?<br>If yes: | □ Yes □ No |
| i. Could you develop a case study? | □ Yes □ No |
| ii. Do you use, or have you used IHME GBD data? | □ Yes □ No |
| **B. BoD support required for:** | |
| Please indicate: | |
| i. Methodology (i.e., method choice, estimation of BoD estimates, etc.) | □ |
| ii. Practical exercises with test data | □ |
| iii. Interpretation of BoD data | □ |
| iv. Communication of BoD data for policy and planning purposes | □ |
| v. Others, please specify: | □ ________ |
| **C. Data environment** | |
| Is data from different sources (i.e., disease registries, surveys, electronic health records, vital statistics, etc.) accessible? | □ Yes □ No<br>Please comment to provide some additional information (if necessary): ____________ |
| Please indicate the level of data coverage: | □ At National level<br>□ At Subnational level<br>□ At Metropolitan level |
| **D. Existing capacity** | |

| | |
|---|---|
| Please indicate the available workforce: | □ Epidemiologist<br>□ Public health expert<br>□ Data scientist<br>□ Statistician<br>□ Programme developer<br>□ Others, please specify: _____ |
| Please indicate the required workforce: | □ Epidemiologist<br>□ Public health expert<br>□ Data scientist<br>□ Statistician<br>□ Programme developer<br>□ Others, please specify: _____ |
| **E. BoD toolkit** | |
| Please provide short description of the nature of toolkit, whether it should be about estimation methods or guidelines or case studies, etc. | |
| **F. Governance** | |
| Please indicate the institution responsible for performing BoD studies (if already in place) or who would be responsible: | □ Public health institutes<br>□ Research institutes<br>□ Academia<br>□ Government<br>□ Others, please specify: _____ |

33
