# Supplementary material for: Recommendations to plan a national burden of disease study
Source: Arch Public Health. 2021 Jul 7;79:126. doi: 10.1186/s13690-021-00652-x (PMC8262070; doi:10.1186/s13690-021-00652-x)
Supplement: Supplementary file 2 — Additional file 2. It describes the experience of countries performing their own burden of disease study as a case study report. [file 13690_2021_652_MOESM2_ESM.pdf]

Additional file 2

Narrative overview of National Burden of Disease studies: A case study report

Table of Contents

Executive summary 3
Key points 3
I. Background 4
II. Objectives 4
III. Methodology 4
IV. Results 4
A. Belgian National Burden of Disease Study (BeBoD) [1] 5
B. Dutch Burden of Disease Study [2] 6
C. German National Burden of Disease Study [3] 8
D. Scottish Burden of Disease Study (SBoD) [4] 11
V. Discussion 16
VI. Conclusions 16
References 17
Appendices 18
A. Appendix1 18

Executive summary

Background: The Global Burden of Disease study, as carried out by the Institute for Health Metrics and Evaluation, produces comparable estimates of mortality, morbidity and disability in the Member States. However, the technical approach used for the GBD study is complex, both in concept and in applications. There are many methodological choices to be made e.g., regarding reference population, redistribution of ill-defined or invalid causes of death, morbidity estimates, life tables, disability weights, which can have an enormous influence on the study's results. The Joint Action has emphasized the potential role of BoD approaches and support countries interested in developing a BoD study but lacking specific expertise to develop their capacity. The main objective of the narrative overview was to provide guidance to those Member States who are planning to perform their own BoD study with particular regard to available data sources, methodological aspects and the implications of BoD data in policy development. Moreover, this overview will support to learn from their experience.

Methodology: The InfAct project partners who are performing their own BoD studies (i.e., Belgium, Germany, The Netherlands and Scotland), were asked to provide an overview of key elements such as data sources used, methodological approaches applied, methodological challenges and related solutions, implication of BoD estimates in health policy and perspectives.

Results: The narrative overview of national BoD studies highlighted important information about the rationale of a study defined, data sources used, choice of a standard population, choices of various methodological approaches, methodological challenges and related solutions, some implications of BoD estimates in health policy and perspectives in the context of four European countries.

Conclusions: This overview highlights the important aspects of performing national BoD studies to support local, subnational and national health policies systematically, generating comparable estimates based on best available local data. The European countries who are planning their national BoD studies, it is important to learn from Belgian, Dutch, German and Scottish experience.

Key points

- The burden of disease approach supports local, regional and national health policies systematically, generating comparable estimates based on best available local data.

- It is important for European countries who are planning their national BoD studies to understand following aspects:

    1. Why should a country want to perform a BoD study?
    2. Choice of a standard population
    3. What methodologies are available and the rational choice of methodological approaches?
    4. What are the benefits of performing a BoD study?

I. Background

The InfAct (Information for Action) is a joint action of Member States aiming to develop a more sustainable EU health information system through improving the availability of comparable, robust and policy-relevant health status data and health system performance information (https://www.inf-act.eu/). InfAct gathers 40 national health authorities from 28 Member States. This Joint Action has proposed the further development of composite indicators to monitor a) burden of disease measures, healthy life expectancies, population attributable fractions due to potentially preventable risk factors at national and subnational levels and b) preventable death based on morbidity indicators related to health system performance and to provide actionable population health information across Europe. Therefore, the Joint Action has emphasized the potential role of BoD approaches and support countries interested in developing a BoD study but lacking specific expertise to develop their capacity. The Global Burden of Disease study, as carried out by the Institute for Health Metrics and Evaluation, produces comparable estimates of mortality, morbidity and disability in the Member States. However, the technical approach used for the GBD study is complex, both in concept and in applications. There are many methodological choices to be made e.g., regarding reference population, redistribution of ill-defined or invalid causes of death, morbidity estimates, life tables, disability weights, which can have an enormous influence on the study's results.

II. Objectives

The main objective of the narrative overview was to guide those Member States who are planning to perform their own BoD study with particular regard to available data sources, methodological aspects and the implications of BoD data in policy development. Moreover, this overview will support to learn from BoD experience of Belgium, Germany, The Netherlands and Scotland.

III. Methodology

The InfAct project partners who are performing their own BoD studies (i.e., Belgium, Germany, The Netherlands and Scotland), were asked to provide an overview of key elements such as data sources used, methodological approaches applied, methodological challenges and related solutions, implication of BoD estimates in health policy and perspectives. We developed a document enlisting the key elements to report and shared with partners to provide the narrative overview accordingly (Appendix 1).

IV. Results

We received four narrative overview from following countries: Belgian National Burden of Disease Study, German National Burden of Disease Study, Dutch National Burden of Disease Study and Scottish National Burden of Disease Study.

A. Belgian National Burden of Disease Study (BeBoD) [1]

The rationale of the BoD study

The main goal of public health policy is to protect and promote population health. Therefore, understanding which diseases pose the greatest threat to health and wellbeing is crucial. Estimates on the burden of disease in Belgium are available from sporadic international and national efforts. However, if disease burden were to support health policy in Belgium, a more systematic approach is required, generating comparable estimates rooted in recent, local data.

Given the need for disease burden estimates to guide decision-making processes within the health sector and the limitations of the currently available burden estimates, Sciensano has taken the lead in launching a Belgian National Burden of Disease Study (BeBoD), which aims to establish a coherent framework for routinely quantifying the burden of disease in Belgium using the DALY metric. The project will ensure ownership and sustainability, embedment within the local context, methodological flexibility and comparability, and capacity building.

Date of start and completion of first BoD study

This BoD study was started on 01/04/2016 and the first set of estimates is expected in the course of 2020.

Data sources used

BeBoD relies on all available routine data sources to estimate the disease burden in Belgium and its regions such as vital statistics (population size, mortality and causes of death), health insurance data, sentinel GP networks, hospital discharge data, health Interview survey, food consumption survey, Belgian Cancer registry, etc.

In addition to routine data sources, BeBoD integrates information from scientific literature and the GBD study as well.

Type of data sources (i.e., linkage at an individual level or aggregated data or both types of data)

In Belgium, there are very few, and limited, routine linkages between data sources. Most estimates will therefore rely on unlinked, aggregated data.

Data accessibility at national, subnational, metropolitan or municipalities' levels

BeBOD aims to quantify burden at national and subnational level, i.e., at the level of the three regions (Brussels, Flanders, Wallonia). All data are available at subnational level.

Quality of underlying data used (i.e., completeness, accuracy, consistency, duplication, etc.)

Every data source has specific limitations. Evaluation of data sources for disease prevalence estimates is done in conjunction with the Eurostat pilot project on diagnosis-based morbidity statistics.

Choice of a standard population: The 2013 European standard population (ESP2013)

Methodological choices used to estimate YLL/YLD:

The main methodological steps involved in the production of BoD estimates (YLL/YLD) are as follows:

- **Years of Life Lost (YLL):** Calculation of YLL at individual level, mapping of ICD-10 codes to GBD cause list and redistribution of ill-defined and invalid ICD-10 codes
- **Years Lived with Disability (YLD):** Prioritisation of diseases, definition of national best estimate for disease prevalence and definition of disease model

Methodological challenges and related solutions

- ***YLL:*** main challenge is related to redistribution of ill-defined and invalid ICD-10 codes. GBD methods are difficult to reproduce because key input data are not publicly available. Choices need to be made regarding method of choice: fixed target distribution vs multiple cause-of-death analysis
- ***YLD:*** main challenge is related to identification of national best estimate for disease prevalence, since a) there is no national health database in Belgium, b) every data source has its limitations, and c) it is not always easy to match the case definitions (implicitly) used in the GBD disease models.

Outcomes/Estimates [interpretation of some main results, level of estimation (i.e., national, subnational or metropolitan levels]

Estimates will be made available by sex, broad age group, and region.

Implications of BoD estimates in health policy

Not yet available.

Perspectives

Next steps will include further fine-tuning of current estimates, extension of list of included causes and risk factors, inclusion of socio-economic status, integration of a forecasting framework and integration of a health impact assessment framework.

B. Dutch Burden of Disease Study [2]

The rationale of the BoD study

The Dutch National Institute for Public health and the Environment provides insights into the most important current and future challenges for public health and health care in the Netherlands. These insights are the basis for the National Health Policy Memorandum of the Ministry of Health, Welfare and Sport and for local public health policy. Next to indicators as, for example, occurrence of diseases and health expenditures, burden of disease metrics are of valuable input to policy makers. Burden of Disease estimates are already included in these

reports since 1997. Over these last two decades of reporting burden of disease, methodology and underlying data quality have been improved, and the application has been broadened, for example towards environmental and occupational health.

Date of start and completion of first BoD study

The first BoD was initiated in 1995 and ended in 1997 (first study).
Ever since, this BoD study was taken further, and published in the different Public Health Status reports (2002, 2006, 2010, 2014 and 2018). Currently RIVM is working on an update to be published in 2020.

Data sources used

The Dutch BoD uses input from many different sources such as basic demographic statistics (population size, overall mortality), causes of death registration, GP registration data (1.6 million records), Dutch Cancer registration, Injury Information System, various health surveys (e.g. Adults Health Monitor 450 thousand records), health insurance data, hospital data and virological weekly reports (surveillance).
In addition, we integrates information from scientific literature and the GBD study.

Type of data sources (i.e., linkage at an individual level or aggregated data or both types of data)

In the Netherlands, different types of data are available. Both at aggregated level and individual level.

Data accessibility at national, subnational, metropolitan or municipalities' levels

Current Dutch BoD studies have bene done place at the national level. Currently we are undertaking a subnational breakdown, at municipality level (350 municipalities).

Quality of underlying data used (i.e., completeness, accuracy, consistency, duplication, etc.)

In general, the quality of the data are adequate, though limitations in use and interpretation have to be taken into account. Especially with survey data, we see variations that are not always the reflection of variation in reality but also measure artefacts. Secondly, the improvement of data quality and expansion of data collection through the years can result in trend data that might not always be consistent over time.

Choice of a standard population: The 2013 European standard population (ESP2013)

Methodological choices used to estimate YLL/YLD:

The main methodological steps involved in the production of BoD estimates (YLL/YLD) are as follows:

- **YLL:** Calculation of YLL based on Dutch Unabridged Life tables (99+ as open ended) distinguished by men/women, mapping of ICD-10 codes to RIVM cause list of 101 disease groups and no redistribution of ICD-10 codes.

- **YLD:** Prevalence estimates for 82 diseases, apply Disability Weights (based on Dutch DW study), correction for multimorbidity (independent occurrence, up to combinations of 5 diseases) and estimation of YLDs for remaining diseases in ICD chapters, not included in the list of 82 diseases.

Methodological challenges and related solutions

- **YLL:** The main challenges are to be flexible in using different life tables to be able to compare results and to include different options to redistribute ill-defined and invalid ICD-10 codes to analyse their effect on the results.
- **YLD:** The main challenges are to have all estimates at population level (now we still have some diseases only at the registration level), to correct for multimorbidity using dependent relation between different diseases and to include national severity distributions based on different proxies for the mild-moderate-severe states.
- **Risk factors:** The main challenges are to including more determinants (more distal, more social/wider determinants) and to improve correction for common end points (now multiplicative, independent).

Outcomes/Estimates [interpretation of some main results, level of estimation (i.e., national, subnational or metropolitan levels]

Estimates are available by sex, broad age group, by disease, by risk factor and DALY component.

Implications of BoD estimates in health policy

Dutch BOD data are used widely to inform and support national and local health policy and planning. It is an important input for the National Public Health memorandum of the MoH. Next, other ministries are using the outcomes as well (mostly risk factor outcomes)

Perspectives

Next steps will include improving current estimates (more diseases, more risk factors), improving the future projections of BoD, including uncertainty and breakdown of BoD at municipality level.

C. German National Burden of Disease Study [3]

The rationale of the BoD study

The growing interest and use of the burden of disease approach led to the initiation of the project Burden of disease in Germany at the national and regional level (BURDEN 2020). BURDEN 2020 responds to challenges that are emerging within the German health system. The current attempts to implement health reforms are facing challenges ensuring that health care planning becomes more closely geared to the morbidity and disability patterns of the population and more responsive to regional needs. The German health system currently lacks a decisive tool that could be used to meet these demands: whereas sub-national analyses of prevalence or incidence of single diseases is available, a comprehensive, noninterest-guided

presentation of the burden of disease is not yet available. The data used by the Global Burden of Disease study for Germany is partly incomplete, and there is no regional stratification available. BURDEN 2020 aims to use the best data available for burden of disease calculation in Germany. It is based on the assumption that ranking individual diseases and risk factors according to the impact that they have on population health would provide a very useful tool to policy makers and other public health stakeholders. Furthermore, the burden of disease approach is presumed to be sensitive to sub-national differences and viewed as having the capacity to generate immediate benefits for regional health policy planning, evaluation and implementation.

Date of start and completion of first BoD study

BURDEN 2020 started in April 2018 and will continue until End of March 2021. First results on YLL in Germany are to be expected for publication as from mid-2020.

Data sources used

German BoD study use following data sources: Population statistics (e.g. mortality, life expectancy), causes of death registration (one underlying cause only), claims data (combining in- and out-patient records, information on medication, inscriptions into disease management programs, etc.), national health interview and examination surveys (for the estimation of risk factor prevalence), national survey designed to estimate pain disorders (back and neck pain, headache disorders) and sequelae for BURDEN 2020, national cancer registry data, national statistics on road injuries and data on population exposure to environmental risks.

Type of data sources (i.e., linkage at an individual level or aggregated data or both types of data)

Most data are accessed at an individual level in order to apply the disease specific concepts that were developed for the GBD study. No data linkage will be conducted.

Data accessibility at national, subnational, metropolitan or municipalities' levels

Causes of death registration and claims data as the main data sources are accessible at municipality level (> 400 municipalities in Germany). However, reporting of BoD estimates will be done at the level of the spatial planning regions (> 90). For survey data, a methodology for small area estimation will be developed in order to obtain regional estimates. Risk attribution will mainly be reported at the national and federal state level.

Quality of underlying data used (i.e., completeness, accuracy, consistency, duplication, etc.)

Causes of deaths registration is a complete data source but contains a considerable proportion of ill-defined codes (> 20 %). Claims data are a complete data source for medically treated morbidity. It lacks information on undiagnosed morbidity and is prone for insurer bias since the claims data used for BURDEN 2020 covers a specific sub-population of about 30 million people (whole population > 80 millions). A methodology correcting for insurer bias will be applied for the estimation of prevalence. Nevertheless, for some diseases with a high level of untreated morbidity (e.g. pain disorders) the use of claims data would lead to an underestimation of the BoD. For pain disorders, we try to fill this data gap with a

representative survey tailored at estimating prevalence and sequelae in line with the BoD concept.

Choice of a standard population: The 2013 European standard population (ESP2013)

Methodological choices used to estimate YLL/YLD
Following are the main choices used:

- **YLL**: Calculate YLL for all GBD causes using German life tables, redistribute ill-defined causes of death
- **YLD**: Prioritisation of diseases for YLD/DALY, measure prevalence and sequelae in claims data using disease specific definitions from best available data from Germany and use GBD disability weights

Methodological challenges and related solutions
YLL

- Ill-defined codes were corrected using proportional redistribution. Redistribution packages provided by IHME were used in order to improve the guiding proportions in a step-by-step approach.
- For the estimation of YLL, we used German lifetables. We created a synthetic life expectancy table using the age specific value on a federal state level with the highest observed life expectancy as a standard for both genders in all regions.

YLD/DALY

- We calculate YLD and DALY for a selection of 19 diseases (> 50% of the German DALY according to GBD)
- If available, we will measure severity distributions (sequelae) for these causes with German data
- If not available we will use GBD global severity distributions
- We will use GBD disability weights to calculate YLD
- We are currently developing a methodology for comorbidity adjustment in line with the GBD methodology

Risk factors

- We calculate risk attribution for selected risk factors
- Risk attribution will be calculated on a federal state level (n=16)
- At the example of one or two risk factors we will try to calculate risk attribution at the level of the spatial planning regions
- We will use risk outcome pairs provided by IHME

Uncertainty
We are currently developing a methodology to estimate uncertainty for YLL, YLD and DALY.

Outcomes/Estimates
Cause specific outcomes will be made available by age, sex and region (> 90 spatial planning regions). Moreover, associations between regional social deprivation and BoD will be estimated.

Implications of BoD estimates in health policy

Not yet available

Perspectives

It is important to disseminate further the results of the pilot project by creating reporting formats that are tailored for different target groups and to further develop adequate visualisation tools. If BURDEN 2020 continues to be financed after the end of the pilot project, we will increase the number of causes, sequelae and risk factors. It is also planned to extend the database to allow for trend analyses and eventually forecasts. Further considerations are to use the BoD data more for health impact measurement.

D. Scottish Burden of Disease Study (SBoD) [4]

The rationale of the BoD study

Following are the main objectives of SBoD: 1. to provide comprehensive data on health needs to support rational resource allocation using all available data sources in Scotland, 2. to identify inequalities in the burden of disease across sub-populations and socio-economic groups, 3. to analyse the contribution to this burden of selected risk factors, 4. to provide epidemiological information against which to compare the relative impacts of interventions in reducing the burden of disease and to inform economic evaluation of those interventions, and 5. to help address the future challenges posed by the ageing of the population, changes in disease and risk factor patterns, and the increasing costs of health services through the production of projections of the disease burden.

Research Questions:

- What is the relative contribution of major disease groupings and of major risk factors to the overall disease burden for Scotland?
- What are the connections formed between disease burden and environmental/ lifestyle risk factors?
- Do geographical regions or socio-economic groups within Scotland have specific patterns of disease burden or of risk factors and what are the implications for resource allocation and cost-effectiveness of interventions?
- Can basic projections of disease burden be used as a guide to potential future demand for NHS services?

Date of start and completion of first BoD study

- The first national BoD study was started on 01/08/2013.
- July 2017, published the first of national results from the SBOD study.

- July 2018, updated national burden estimates with new analysis and supporting data visualizations on assessing the extent to which health inequalities were associated with the burden of disease in Scotland.
- July 2019, produced burden estimates at the following sub-national levels: regions (3), NHS boards (14) and local authorities (32).Date of completion/end: study is ongoing.

Data sources used

The Scottish Burden of Disease study relies on all available routine data sources to estimate the disease burden in Scotland at a national and local level. Following data sources are used: Vital statistics (population size, mortality records and causes of death), general and Psychiatric Hospital Stays/day cases (including intensive care/high dependency stays), outpatient attendances, emergency department attendances, maternity, birth records and Neonatal Care, GP consultations sample, GP Disease Registers, community prescriptions, dental treatments, surveillance of communicable disease, national health interview survey (Scottish Health Survey), Scottish cancer registry, diabetes register and learning disability statistics
In addition to routine data sources, SBoD integrates information from scientific literature and the GBD study.

Type of data sources (i.e., linkage at an individual level or aggregated data or both types of data)

For the majority of causes of disease or injury in the SBOD study, estimates have been made using patient-level data sources, which contain a patient Community Health Index number, which was harnessed to enable the linkage of datasets. Where patient-level data was unavailable estimates rely on unlinked aggregated data.

Data accessibility at national, subnational, metropolitan or municipalities' levels

All data are available at national (5.4 million people) and sub-national level, including regional health authorities (average 1.8 million people), NHS Boards (average 386,000 people) and local authorities (average 169,000 people).
National data include estimates at socio-economic level. YLD estimates for local areas are based on expected results only and have been modelled using the national age, sex and deprivation morbidity rates generated as part of the SBOD 2016 study.

Quality of underlying data used (i.e., completeness, accuracy, consistency, duplication, etc.)

Mortality

In Scotland, deaths are recorded on the National Records of Scotland (NRS) register of deaths. Every death in Scotland must be certified by a doctor who completes a form called a Medical Certificate of Cause of Death (MCCD). The accuracy and quality of MCCD is routinely monitored. The information in the MCCD was used to extract the age, gender, and postcode of residence and the underlying and contributory cause of death, for each individual death in Scotland.

Morbidity

In Scotland, there is a wide-range of electronic health records with nationwide coverage relating to contacts with healthcare settings across a range of both primary and secondary care services. There are also several other rich sources of data e.g. disease registries or surveys, which can be used to determine estimates of morbidities. For the majority of causes of disease or injury in the SBOD study, estimates have been made using data sources, which contain a patient Community Health Index (CHI) number, which was harnessed to enable the linkage of datasets. The advantage of using datasets that contained CHI numbers were that the CHI enabled us to derive information relating to the patient demographics such as age, gender or socioeconomic status. The presence of a CHI number also allowed us to censor patients from estimates of morbidity when they died, and furthermore, allowed us to search across several datasets to ensure that our estimates of morbidity were as extensive as possible. For example, we linked records from unscheduled care services with hospitalisations episodes data for a more comprehensive assessment of the burden of injuries.

Choice of a standard population: The 2013 European standard population (ESP2013)

Methodological choices used to estimate YLL/YLD

The main methodological steps involved in the production of BoD estimates (YLL/YLD) are as follows:

- **Years of Life Lost (YLL):** Allocation of Scottish morality data to abridged GBD disease classification, redistribution of ill-defined and invalid ICD-10 codes and calculation of YLL at individual level using Scottish life tables
- **Years Lived with Disability (YLD):** Definition of national best estimate for disease prevalence, alignment of GBD disease model with data-source, apply GBD (or Scottish specific where available) severity distributions, apply disability weights, co-morbidity correction and calculation of YLD at individual level

Methodological challenges and related solutions

YLL

Choice of life table in burden of disease studies

SBOD approach differs to that of GBD, which used an aspirational life table with a higher life expectancy than currently observed in Scotland and assigns the same life expectancy to both men and women. We opted to use a Scottish-based life table to reflect the true circumstances regarding mortality that we are observing as a nation.

Redistribution of ill-defined deaths

GBD methods are difficult to reproduce because key input data are not publicly available. SBoD created their own process for allocating IDDs: Firstly, allocations were made on the basis of drug-related deaths and injuries in the second contributory cause of death position. The next step was then to assign deaths causes of disease and injury by using the information

pertaining to all contributory causes of death on the death certificate. See Grant et al 2018 in The Registrar General's Annual Review of Demographic Trends.

YLD

Prevalence estimates

The identification of the best data source to estimate disease prevalence, and alignment with GBD disease models. This requires extensive understanding of data sources available within the country and how the application of the GBD disease model parameters can be used in conjunction with the identified data source.

Severity distributions

Understanding the impact of international severity distributions on estimates of national burden of disease, see Wyper et al 2019 'The impact of worldwide, national and sub-national severity distributions in Burden of Disease studies: A case study of cancers in Scotland'.

Comorbidity adjustment

There are three main differences between SBOD and GBD in terms of comorbidity adjustment. First, SBOD creates a simulation for the population subgroup defined by each five year age groups, gender and deprivation decile stratum. Second, we limit the number of morbidities an individual can have based on the results of Barnett et al. 2012, and third we adjust the disability weight at disease level instead of sequela level.

General

Age standardised rates

Disease rankings based on DALY ASRs are strongly influenced by the choice of standard population. While GBD world standard population offers international comparability, within-country analyses based on DALY ASRs should reflect local age structures. For European countries including Scotland, ESP2013 may better guide local priority setting: see Wyper et al 2019 **'**the impact of World and European Standard Populations in Burden of Disease studies: A case study of Disability-Adjusted Life Years in Scotland', *in press*

Uncertainty intervals

Estimating uncertainty in estimated disease burden is difficult to do, because apart from the large number and disparate nature of the data sources used, information or knowledge about the quality of and potential biases in the data is often limited.

In order to provide a measure of the degree of accuracy and relevance of the estimated disease DALYs to users, a measure of data quality has been developed for the SBoD study. This measure assigns a RAG (Red; Amber; Green) status to each disease or injury indicative of the accuracy and relevance of the estimates. Interpretation of the RAG status can be defined as follows:

- GREEN Highly accurate and relevant: Estimates have been derived using relevant and robust data sources with only a small degree of adjustments performed to the input data.

- AMBER Moderately accurate and relevant: Estimates have been derived using reasonably relevant and robust data sources with only a moderate degree of adjustments performed to the input data.
- RED Uncertainties over accuracy and relevance: Estimates have been derived using less comprehensive or relevant data sources with a high degree of adjustments performed to the input data.

The data quality has been assessed using three following main criteria that are subjectively assessed and each criterion is scored on a scale of 1 to 5:

I. Relevance and accuracy of the data source used to measuring the population of interest
II. Likelihood that the implemented disease model captured the overall burden of disease or injury
III. The relative contribution of ill-defined deaths to YLL, and YLL to DALY.

Outcomes/Estimates [interpretation of some main results, level of estimation (i.e., national, subnational or metropolitan levels]

Estimates are available by sex, age group, at socio-economic status, and at sub-national level (regional, local and by health authority): https://www.scotpho.org.uk/comparative-health/burden-of-disease/sbod-data-2016/ and https://www.scotpho.org.uk/comparative-health/burden-of-disease/sbod-local-2016/

Implications of BoD estimates in health policy

SBOD data used widely to inform and support national and local health policy and planning, for example: Scottish Government Public Health Priorities for Scotland, Chief Medical Officer Annual Report for Scotland, Audit Scotland (Scottish parliament's watchdog for ensuring propriety and value for money in the spending of public funds), Registrar General's Annual report (to the Scottish Parliament), Scottish Government mental health strategy, Scottish Government neurological conditions national action plan, Motion in Scottish Parliament in response to SBOD findings, Petition into full review of mental health service provision, Drug Deaths Taskforce, working with local areas in Scotland through community health and social care partnerships to use SBOD data information to inform needs assessment, health planning and workforce decisions.

Local estimates have been embedded in many of the 31 Health and Social Care Partnership Strategic Needs Assessments for example Clackmannanshire and Stirling, Falkirk, Inverclyde and West Dunbartonshire. BoD estimates are also used in reports of Directors of Public Health reports, such as Fife and Lanarkshire, third sector policy statements, such as Arthritis Research UK.

Perspectives

Next steps will include to explore the impact and value of method choices in burden of disease studies (ongoing work), Scottish Burden of Diseases 2019 (will publish in 2020) – updating of

full burden of disease results, ongoing development of data visualizations to support the dissemination of study findings, projections of disease burden to 2040 (based on population projections and trends) such as 'what if' we could modify exposures (poverty, alcohol, tobacco, obesity) – what would the projections look like then, what would be the primary and secondary interventions, which would likely to have the biggest impact on the projection, which would be the most cost effective interventions and what would be /are the workforce implications?

V. Discussion

The narrative overview of national BoD studies highlighted important information about the rationale of a study defined, data sources used, choice of a standard population, choices of various methodological approaches, methodological challenges and related solutions, some implications of BoD estimates in health policy and perspectives in the context of four European countries. The burden of disease approach supports local, regional and national health policies systematically, generating comparable estimates based on best available local data. Moreover, it contributes to identify inequalities in BoD across sub-populations and socio-economic groups and to support the rational allocation of resources in a country. These case studies used/are using most common data sources such as routinely collected administrative data, vital statistics, disease-specific registries, national health surveys (interview and/or examination), etc. In addition, data from scientific literature and the GBD study is also integrated. These data sources are accessible at national, sub-national/regional and at municipal levels. Common methodological challenges such as redistribution of ill-defined codes, choice of a national life table, disability weights, severity distributions, comorbidity adjustment, risk attribution and age-standardized rates, were reported. As perspectives, following are the steps to further improving the BoD estimates and to establish different frameworks: extend the list of causes, sequelae and risk factors, the inclusion of socio-economic status and integration of forecasting and health impact assessment frameworks.

Recommendations: It is important for European countries who are planning their national BoD studies to understand three following aspects: 1. why should a country want to perform a BoD study, 2. what methodologies are available and the rational choice of methodological approaches and 3. what are the benefits of performing a BoD study.

VI. Conclusions

This overview highlights the important aspects of performing national BoD studies to support local, subnational and national health policies systematically, generating comparable estimates based on best available local data. The European countries who are planning their national BoD studies, it is important to learn from Belgian, Dutch, German and Scottish experience.

References

1. Cornez A, Devleesschauwer B, Renard F: Belgian National Burden of Disease Study: https://www.sciensano.be/en/projects/belgian-national-burden-disease-study. 2016.
2. Melse JM, Essink-Bot ML, Kramers PG, Hoeymans N: A national burden of disease calculation: Dutch disability-adjusted life-years. Dutch Burden of Disease Group. *Am J Public Health* 2000, 90(8):1241-1247.
3. von der Lippe E, Rommel A, Plass D, Schröder H, Ziese T: The German national Burden of Disease study BURDEN 2020. *European Journal of Public Health* 2018, 28(suppl_4).
4. Grant I, Mesalles-Naranjo O, Wyper G, Kavanagh J, Tod E, Fischbacher C, McCartney G, Stockton D: Burden of Disease in Scotland: https://www.nrscotland.gov.uk/files//statistics/rgar-invited-chapter/rgar17-invited-chapter.pdf. 2017.

Appendices

A. Appendix1

Member states provided a *narrative overview* of their current experience conducting their own BoD studies according to the following elements:

1. The rationale of the BoD study
2. Date of start and completion of first BoD study
3. Data sources used (i.e., vital statistics, electronic health record, cohort studies, administrative data sources, disease registries, census data, mix of different available sources, etc.)
4. Type of data sources (i.e., linkage at an individual level or aggregated data or both types of data)
5. Data accessibility at national, subnational, metropolitan or municipalities levels
6. Quality of underlying data used (i.e., completeness, accuracy, consistency, duplication, etc.)
7. Choice of a standard population
8. Methodological choices used to estimate YLL/YLD:
   What are the main methodological steps involved in the production of your estimates? Some of the technical steps are enlisted in *table 1 below*. You may use that table to provide technical detail. If some technical steps are not mentioned in *table 1* and you have adopted different steps, please feel free to add them in this overview.
9. Methodological challenges and related solutions
10. Outcomes/Estimates [interpretation of some main results, level of estimation (i.e., national, subnational or metropolitan levels]
11. Implications of BoD estimates in health policy (impact on health policy)
12. Perspectives: What do you intend to do now that you have BoD estimates? Are you looking into greater refinement of your methodology? Use of more data sources? Increasing the number of causes and conditions?
