# Supplementary material for: Recommendations to plan a national burden of disease study
Source: Arch Public Health. 2021 Jul 7;79:126. doi: 10.1186/s13690-021-00652-x (PMC8262070; doi:10.1186/s13690-021-00652-x)
Supplement: Supplementary file 3 — Additional file 3. It describes the study results comparing the country health profiles and national health statistics and inputs from different countries. [file 13690_2021_652_MOESM3_ESM.pdf]

Additional file 3 : Comparison of country health profiles (i.e., GBD metric) with national health statistics in European Countries

Table of Contents

Executive summary 3
Key points 4
I. Background 5
II. Objectives 5
III. Methodology 5
IV. Results 6
A. Comments on country health profiles and potential differences in estimates/trends 6
1. Life expectancy [LE] 1990 – 2017 (Figure 1) 6
2. Age-standardized mortality rates by top ten conditions 1990 – 2017 (Figure 2) 7
3. Age-standardized Years of Life Lost [YLL] 1990 – 2017 (Figure 3) 8
4. Age-standardized Years Lived with Disability [YLD] 1990 – 2017 (Figure 4) 9
5. Age-standardized DALYs 1990 – 2017 (Figure 5) 9
6. Age-standardized DALYs due to known risk factors 1990 – 2017 (Figure 6) 10
7. Annual change in age-standardized DALYs attributable to top ten health conditions 1990 – 2017 (Figure 7) 11
8. Healthcare Access and Quality Index (HAQ) based on amenable mortality 2017 (Figure 8) 12
B. Additional data sources used by individual countries for national health reporting . 12
V. Discussion 14
VI. Conclusions 16
VII. References 16
VIII. Additional files 17
A. Additional file 1 17
B. Additional file 2 22
C. Additional file 3 24

Executive summary

Background: The country profiles provide a measure of priority health conditions and risk factors, a summary breakdown of major causes, and an appreciation of health sector performance, according to the GBD methodology. The main objectives of this study were to compare the way GBD identifies top priorities in health outcomes, risk factors and health sector performance, with a country's assessment based on their national health reporting and to identify the potential differences in estimates due to different data sources and methods used by countries producing their own BoD estimates.

Methods: Using the 'standard' GBD metrics available in the GBD 2017 study, we have produced a series of country health profiles of European countries. All charts have been produced using the same R code. We uploaded them on an interactive website created only for 'Country Health Profiles' and shared with 30 countries with an electronic link. The survey participants were from public health institutes and were involved in burden of disease activities in their country.

Results: The response rate was 76 % (23/30). This comparison highlighted that in the majority of countries the priority health conditions and risk factors based on national health statistics correspond to the IHME ranking of health conditions. However, in some European countries, certain differences were identified such as in France, the trend of musculoskeletal disorders, mental health and neurological diseases to the disability has changed since 2010 and occupational risks are underestimated by IHME. In Romania, mortality rates for cardiovascular diseases and cancer are under-estimated by IHME as compared to national statistics and premature mortality for cardiovascular diseases are over-estimated by IHME. In Serbia, mortality rates for cardiovascular and respiratory diseases were under-estimated by IHME as compared to the Serbian BoD study in 2000. In Scotland, the mortality rates due to substance use have higher numbers in the national death register compared to those used by IHME. Some countries reported additional data sources used by the individual country for national health reporting not enlisted in IHME list. The countries who calculate their own BoD estimates mentioned that choice of a standard population and the use of different methodological choices could influence the estimates.

Conclusions: Our results highlighted some variations in ranking of health conditions and risk factors by comparing IHME estimates with individual countries assessments based on their national health reporting. Very few countries have a complete BoD assessment, i.e. comprehensive, updated, and repeated over time to identify trends. For countries who develop their own BoD study, the inter-country comparability remains an issue due to different data sources, choice of a standard population and different methodological approaches applied.

Keywords: Burden of disease; Mortality; Morbidity; DALYs; Risk factors; GBD metrics

Key points

- The importance of key aspects such as good quality data sources, choice of a standard population and different methodological choices used to calculate BoD estimates, can influence the estimates and the ranking of diseases and risk factors.

- Building analytical capacity and awareness of different methodological approaches are necessary for developing national BoD studies.

- The comparability and consistency of estimates across diseases are essential to estimating BoD estimates and can strongly influence the local, national and European levels policy decisions.

- A European data infrastructure is required, to support development of the European BoD estimates that would facilitate sharing similar data sources and common methods used to calculate BoD estimates at EU-level.

I. Background

The country profiles provide a measure of priority health conditions and risk factors, a summary breakdown of major causes, and an appreciation of health sector performance, according to the GBD methodology [1]. This approach highlights the usefulness and possible applications of a standardized, comprehensive methodology in Burden of Disease assessment and allows a standardized comparison with European peer countries. However, most of the European countries do not produce GBD-metrics (YLLs, YLDs, DALYs). Therefore, we intend to compare the ranking of priorities in diseases and risk factors, using GBD metrics (as provided by the Country Health Profile developed using the Institute for Health Metrics and Evaluation methodology), to the ranking of a country's standard metrics (mortality, morbidity rates, risk factor prevalence, etc.). For example, the national ranking of a given country could be based on risk factor prevalence showing that smoking is its main health problem, whereas GBD-metrics (i.e., DALYs attributable to risk factors) may show that alcohol is more important. No attempt has been made to provide a detailed background narrative to the findings of country health profiles.

II. Objectives

The main objectives of this study were to compare the way GBD identifies top priorities in risk factors, health outcomes and health sector performance, with a country's assessment based on their national health reporting and to identify the potential differences in estimates/trends due to different data sources and methods used by countries producing their own BoD estimates.

III. Methodology

Using the 'standard' GBD metrics, we have produced a series of country health profiles. All charts have been produced using the same R code, as an example of Public Health England's Reproducible Analytical Pipelines procedures. All data used are publicly available, at https://vizhub.healthdata.org/gbd-compare/ and http://ghdx.healthdata.org/gbd-results-tool. Almost, 80,000 different data sources were used to produce these country health profiles. The information on data sources can be found here: http://ghdx.healthdata.org/gbd-2017/data-input-sources. After producing the charts for country health profiles of European countries, we uploaded them on an interactive website created only for 'Country Health Profiles' (https://espaces.santepubliquefrance.fr/espace_projets/Accueil/gbd). A username and password was provided to each country, accessing this website of country health profiles and uploading their comments.

A brief description of GBD metrics was used to describe country health profiles with an example of the England country health profile (additional file 1) and shared with the participants. An invitation email was sent on February 5, 2020 to 30 European countries to compare their country health profile with their country's current assessment based on their

national reporting. The survey participants were from public health institutes and were involved in burden of disease activities in their country. The abbreviation of the countries' names are reported in additional file 2. One following reminder was sent to the non-respondents to submit their comments on June 8, 2020. The 30 country health profiles are reported in additional file 3.

IV. Results

We received the comments from 23 countries with a response rate of 76% (23/30). These results are reported under following sections: A. Comments on country health profiles and the potential differences in estimates/trends and B. Additional data sources used by the countries for national health reporting.

A. Comments on country health profiles and potential differences in estimates/trends

We described the comments on country health profiles by taking into account the IHME estimations and the national health statistics of each country. Some countries mentioned potential underlying factors explaining the differences between GBD estimates and their country results. The countries did not calculate these estimates to compare with their national health statistics.

Here is the summary of country health profiles including life expectancy, mortality, premature mortality, morbidity, DALYs, risk factors, DALYs breakdown and Health Care Access and Quality Index in European countries:

1. Life expectancy [LE] 1990 - 2017 (Figure 1)

The observed life expectancy estimates are in line with national estimates in BE, DE, FI, FR, HU, IT, LV, NO, RO, SI, and SC. For example *in Belgium*, according to the national estimates of 2018, the observed LE was 81.5Y (both gender), 83.7 (*F), 79.2 (*M), whereas according to IHME estimates in 2017, the observed LE was 81.4 (both gender), 83.8 (F), 78.9 (M). *In Norway*, according to the national estimates of 2017, the observed LE was 82.6Y (both gender), 84.3 (*F), 80.9 (*M), whereas according to IHME estimates in 2017, the observed LE was 82.3 (both gender), 84.2 (F), 80.5 (M).

The observed LE estimates are better than expected life expectancy in all respondent countries except for, BG, EE, FR, LV, LT, RO and SK. Latvia and Lithuania mentioned some underlying factors influencing the drop in observed LE such as collapse of Soviet Union in 1990, economic crisis in 2008, socio-economic and health inequalities.

Potential difference in LE estimates/trends: *Serbia*, reported that observed LE showed slight/moderate differences between national and IHME estimates in some years, such as in 2000 where the observed LE estimated by their national health authority was 74.81 (F) and

69.2 (M), whereas IHME estimated 75.5 (F) and 68.9 (M). *In Spain*, according to the national health statistics, the trends for observed LE were stable during 2016-2017, whereas according to the IHME, this indicator has a tendency to increase.

** F: Female, M: Male*

2. Age-standardized mortality rates by top ten conditions 1990 - 2017 (Figure 2)

According to IHME, the mortality rates have shown a marked decrease in cardiovascular diseases among all European countries since 1990. It represents the largest share of the burden of mortality in the following countries: AT, BE, BG, CY, CZ, DE, EE, FR, FI, HR, HU, LV, LI, MT, PL, PT, RO, SK, SI, SRB and SW. In some countries (DK, ENG, ES, FR, IR, IT, NL, NO), due to sharp decrease in cardiovascular mortality rates, cancer has become the most contributing factor to the burden of mortality. In most of the European countries, the ranking of IHME estimates of age-adjusted mortality rates by top ten conditions more or less match the national estimates.

Contrary to the sharp decrease in mortality rates of cardiovascular diseases, there is a slow decrease in cancer mortality rates across all European countries. Despite improved primary care, better treatments, new diagnostic technology, screening programs, promotion of healthy life style, etc., the mortality rates of cardiovascular diseases and cancer are still contributing most to the burden of diseases in all European countries.

IHME estimates also highlight that the neurological disorders (i.e., Alzheimer's, dementia, etc.) are the third most contributing factor to the burden of mortality in all European countries except in Cyprus, where mortality rates due to diabetes mellitus is the third most contributing factor to the burden of mortality. Since 1990, the mortality rates for neurological diseases were nearly unchanged in all European countries, which could be explained due to lack of new treatments and ageing population.

Potential difference in mortality estimates/trend: *In Belgium*, according to national estimates developed using the SPMA procedure (standardized procedure mortality analysis: https://spma.wiv-isp.be/SitePages/Home.aspx) in 2016, cardiovascular diseases are ranked as the top contributing factor to the burden of morality by using the European standard population as a reference population. However, by changing the reference population from European Standard Population to the World Standard Population, this ranking changes and cancer mortality rates have become the first contributing factor to the burden of mortality as used by IHME. The estimates calculated by SPMA do not take into account the redistribution of ill-defined codes. *In Estonia*, the mortality rate for neurological disorders have grown year by year but according to the European standard population the rate for injuries (unintentional and intentional self-harm) are higher than the rate for neurological disorders and there are more respiratory infections than neurological disorders. *In Romania*, an important difference was observed i.e., the mortality rates for cardiovascular diseases are below 400/100,000 according to IHME estimates but according to national health statistics, these estimates are higher, and slightly decreasing from 725 in 1999 to 677 in 2017, respectively. The mortality

rates for cancer are increasing from 173 in 1999 to 233 in 2017 according to national statistics but these mortality rates are less than 200 according to IHME estimates. *In Serbia*, mortality rates for cardiovascular and respiratory diseases were underestimated by IHME as compared to the Serbian BoD study in 2000 due to different methods of estimation. *In Scotland*, the mortality rates in 2013 and 2016 due to substance use had higher numbers in national death register. The reason for this increase was due to use of an expanded set of ICD codes for drug related deaths. *In Spain*, the national health statistics reported an increase in mortality rates of respiratory and neurological diseases between 2010 and 2017 whereas according to IHME these mortality rates are decreasing.

3. Age-standardized Years of Life Lost [YLL] 1990 - 2017 (Figure 3)

According to the IHME estimates, cardiovascular and cancer are the major diseases contributing to the YLL in European countries. The mortality rates of these two health conditions have a decreasing trend from 1990 to 2017. The majority of the countries who do not calculate their BoD estimates, confirm that the trend of cardiovascular and cancer correspond to their national health statistics.

In Romania, during 1990-2017, there is an important difference in premature mortality due to cardiovascular diseases with a decreasing trend from 7000+ to 5000+ in IHME data and a decreasing trend from 5000+ to 3000 according to national statistics. Moreover, the premature mortality estimates for cancer are slightly higher at the beginning of the previously mentioned period.

In Slovenia, the premature mortality is decreasing in Slovenia and in 2018, the main contributor was the cancer premature mortality (44%) and followed by cardiovascular diseases (17%).

Potential challenges to compare YLL estimates: Some countries who are performing their BoD studies mentioned following challenges to compare the national YLL estimates, with the IHME estimated YLL:

- The national YLL estimates are calculated without taking into account the redistribution of ill-defined codes (Belgium). As well a different methodology of redistributing ill-defined codes may be applied (Germany).
- Changing the reference population to World or European Standard Population influences the ranking of diseases contributing to YLL (Belgium, Estonia, Scotland). Belgium and Scotland used the European standard population as a reference.
- The use of national life tables and population LE influences the ranking of diseases contributing to YLL (Estonia, Germany, Scotland).
- Mortality rates for cardiovascular and respiratory diseases were underestimated by IHME as compared to the Serbian BoD studies due to different methods of estimation in 2000 (Serbia).

4. Age-standardized Years Lived with Disability [YLD] 1990 - 2017 (Figure 4)

This is not a common indicator used in the European countries. According to IHME estimates, musculoskeletal disorders, mental health and neurological diseases are the main health conditions contributing to disability in the majority of European countries. In some countries, unintentional injuries are either second (CZ, HU, PL, RO, SK) or third (BG, EE, LT, LV) main contributors to disability (i.e., YLD) until 2017. The overall trend of various health conditions contributing to YLD estimates remained unchanged during 1990 – 2017. It is noteworthy that among all European countries, the trend of musculoskeletal disorders and neurological diseases remains unchanged almost over the last three decades according to the IHME estimates. This has a strong impact on disability and may influence increased use of rehabilitation services, absence from work, sick leave or early retirement.

According to the *Estonian BoD study* in 2015 and 2017, there were top four diseases contributing to the YLDs: 1. Circulatory diseases, 2. Musculoskeletal disorders, 3. Neoplasms and 4. Sense organ diseases. Neurological disorders are often underestimated in Estonia due to poor knowledge of diagnosing Alzheimer's., *Slovenia* reported that according to national health statistics, the diabetes and kidney diseases are the second main contributor to the morbidity and this estimate fluctuate over this period, which may be due to more systematic implementation of prevention programs.

Potential challenges to compare YLD: The countries who are performing their own BoD studies reported that the comparison of national YLDs estimates with IHME is challenging due to following reasons: use of different data sources, different methods used for age-standardized rates, differences in prevalence rates, estimation of country specific severity distributions and duration parameters.

Potential difference in YLD estimates/trend: In France, the evolution of musculoskeletal disorders, mental health and neurological diseases contributing to the disability has changed since 2010 as compared to the IHME trends.

5. Age-standardized DALYs 1990 - 2017 (Figure 5)

DALYs are not yet produced in the majority of European countries; therefore, the comparison with IHME estimates was not possible.

According to IHME estimates in 2017, among 20 countries (AT, BG, CY, CZ, DE, EE, FI, GR, HR, HU, LV, LI, MT, PL, RO, SC, SRB, SK, SI, SW), either cancer or cardiovascular diseases are the top two health conditions contributing to the DALYs. Among 11 countries (BE, DK, ENG, ES, FR, IR, IT, LU, NL, NO, PT), the musculoskeletal disorders are the second most contributing health condition to the DALYs. Among eight countries (CZ, EE, LV, LI, PL, RO, SK, SI), unintentional

injuries are the third most contributing health condition to DALYs. In France and Italy, mental health is the third most contributing health condition to DALYs.

Five countries (DE, FI, HU, IT, SC) mentioned that, according to the national health statistics, some priority health conditions highlighted by IHME are similar to their own national priorities/goals.

In France, there is an increase in some mental health disorders. Between 2010 and 2014, there was a significant annual increase in hospitalizations and ambulatory care of patients with anxiety disorders (3.6% in the rates for females [$p<.001$] and 3.7% in the rates for males [$p<.001$]) and patients with bipolar disorder (2.6% in males [$p=.01$] and 3.4% in females [$p<.001$] in public psychiatric settings. The rate of health care for depressive disorders has not changed over the studied years [2, 3].

Potential challenges when comparing DALYs: Estonia, Germany, Scotland and Serbia who produce their own BoD estimates, mentioned that due to different standard populations and methodological approaches, the direct comparison of their estimates with IHME is not possible.

6. Age-standardized DALYs due to known risk factors 1990 - 2017 (Figure 6)

According to IHME, in majority of the European countries, the contribution of individual risk factors (i.e., 19) to the overall burden (i.e., DALYs) show a steady decrease over this period. According to the 3-category structure of risks used by GBD, the following are the risk factors whose contribution to the DALYs changes slightly over this period:

1. *Behavioural risk factors* including alcohol use, dietary risks, low physical activity, tobacco use and unsafe sex.
2. *Metabolic risk factors* including, high LDL-cholesterol and high systolic blood pressure.
3. *Environmental risk factors* including air pollution.

There are some risk factors, whose contribution to DALYs changes little or not at all over this period in European countries. For example, drug use, intimate partner violence, childhood maltreatment, high body-mass index, high fasting plasma glucose, occupation risks and other environmental risks. *In Scotland*, the drug use has increased over the last decades. In the European region, the overall burden of disease/diseases due to known risk factors has decreased but the relative contribution of a single risk factor within a given year has largely stayed unchanged.

According to the national health statistics, DALYs due to alcohol use are decreasing over this period in Latvia, Lithuania and Poland. *In Latvia* as part of public health framework strategy to reduce alcoholism, following actions have been planned in 2014-2020 and 2020-2022: a gradual increase in excise tax, bans and restrictions to alcohol advertisement and sale.

According to *Lithuania*'s health strategy, the alcohol use is decreasing since 2015. *In Poland*, the main risk factors with the largest share in DALYs are tobacco (21,2%), dietary risks (19,3 %) and metabolic risks like high systolic blood pressure 12,4%; high LDL cholesterol 10,2%; high body-mass index 9,7%, alcohol use 8,9%. *In France*, the overall trend of various risk factors corresponds to the national health statistics. *In Scotland*, the existing knowledge about the contribution of certain risk factors to the overall disease burden confirms that the tobacco use, diet, obesity and other metabolic risk factors are main contributors to overall disease burden.

According to public health strategy frameworks across many European countries, prevention intervention programs help to increase awareness via health campaigns promoting stop smoking, healthy diet, regular physical activities and less alcohol drinking.

Potential difference in risk factor estimates/trend: In France, the trend of occupational risks are underestimated by IHME.

Potential limitation related to method used: The IHME estimates should be interpreted with caution, as individual risk factors may become intermediary factors in a causal pathway. The IHME methodology works with single risk-outcome pairs, which does not take into consideration interactions between risk factors or one risk factor being an intermediary to another one in a pathway.

7. Annual change in age-standardized DALYs attributable to top ten health conditions 1990 - 2017 (Figure 7)

According to the IHME estimates, the annual change in age-standardized DALYs attributable to individual health conditions is progressively declining over time in European countries. Cardiovascular and cancer are the main contributors to the annual decrease in overall DALYs in all European countries and to a lesser extent, unintentional injuries, diabetes, respiratory conditions, substance use disorder and other non-communicable diseases. In some European countries, the contribution of musculoskeletal disorders (DK, ENG, FR, IE) and substance use disorder (FI) to the overall DALYs has increased in 2017.

According to the national health statistics, some European countries (CY, FI, IT, PL, PT, LV, LT) mentioned that the changes in cardiovascular diseases correspond to their national health reporting as reflected by premature mortality estimates (i.e., YLL in figure 3).

Potential difference in attributable DALYs estimates/trend: *In Estonia*, there are quite some differences between the causes of YLLs and YLDs. If we sum them up, then the real problems cannot be observed. *In France*, the trend in interpersonal violence does not reflect the real-time situation. *Germany* mentioned that their national health reporting does not fully reflect these variations. Most of the European countries did not comment on these estimates because DALYs are not commonly calculated at national public health institutes as part of routine activities.

8. Healthcare Access and Quality Index (HAQ) based on amenable mortality 2017 (Figure 8)

According to IHME estimates in 2017, the HAQ index (i.e., a proxy measure of the quality of health care services based on amenable mortality [32 causes of death]) shows that in most European countries, results for circulatory diseases, respiratory diseases and some types of cancers are close to the highest percentile. This reflects the performance of health care services in those conditions amenable to better outcomes where high quality care is available. Improvements in life expectancy are linked to it.

On the other hand, the HAQ index for the treatment of non-melanoma skin cancer (squamous-cell carcinoma) is lower than 60 in following European countries, highlighting the potential issues with diagnosis, treatment and care: AT, BE, CY, DK, EE, ES, HR, HU, LV, LT, PL, PT, RO, SRB, SK, SI.

Some countries mentioned that the HAQ index is a rough proxy measure, useful to compare the performance of health care services across European countries, but that it is of limited use for an in-depth improvement in the delivery and quality of health care services (DE, PL). Due to lack of knowledge on data sources and methods used for amenable mortality estimation, it is difficult to interpret these estimates (HU).

B. Additional data sources used by individual countries for national health reporting

IHME uses a broad range of various types of data sources to calculate GBD estimates. Some countries mentioned that many of these sources are not commonly used in routine calculation of their national health statistics.

Following countries mentioned additional data sources, which are not included in the list of data sources by IHME:

Croatia: Data sources to calculate related estimates of mortality, morbidity and for risk factors used by the IHME are appropriate. A data source that is often used in national statistics and is not included in the list of IHME data sources is EHIS [European Health Interview Survey - 2nd wave].

Czech Republic: Data sources used by IHME often refer to international health-related databases (ECHI, OECD, WHO) to which the Institute of Health Information and Statistics of the Czech Republic contributes. In some cases, more recent data are available than listed. Some results are based on local studies, which may not be fully generalizable to the Czech population. More detailed data are used for national health statistics since the primary data are available within national health registries such as National Cancer Register, National Register of Hospitalized Patients, National Register of Reproduction Health, National Register of Cardiovascular Surgery and Intervention etc. In 2018, National Register of Reimbursed Health Services was

launched, containing individual-level claims data of the whole Czech population (10.6 million) from 2010 to the present, updated quarterly. Nowadays, it is the main source of information on morbidity based on inpatient and outpatient care records including diagnoses and treatment (procedures, medications).

Finland: the IHME data source for mortality data is the WHO mortality database and latest data is from 2015. From National Statistics Finland, more up-to-date information by sex until 2018 can be obtained. Death from 2019 will be published at the end of 2020. The latest morbidity information used by IHME from the Hospital Discharge Register is from 2014. For national calculations, we can obtain more up-to-date information about in- and out-patient hospital visits from the care register. For risk factors such as smoking, hypertension and obesity, the latest questionnaire data used by IHME is from 2014 and health examination data from 2012. Since then, we have had a health examination survey in 2017 and questionnaire-based health surveys annually, latest completed data from 2019. In our national calculations, we can also use data on medical prescriptions and purchases.

France: The following data sources are not included in the list of IHME data sources and are frequently used in national health statistics: surveys (ENRED, Baromètres santé, ESPS), INSERM CépiDC for national mortality data and INCA database for cancer.

Germany: IHME heavily relies on evidence that is published in scientific journals in English language. Thus, with some exceptions like hospital data or causes of death data national data sources are not systematically accessed in order to estimate the burden of disease. In Germany, for instance, both national statistics and survey data on road injuries are accessible at micro data level, are not listed among the data that is used to estimate the burden of road traffic injuries. Some type of data that is important to consider for accurate national estimates, may be difficult to access for foreign research institutions due to data protection regulations (e.g. claims data).

Italy: Most of the data sources used by the IHME to calculate estimates of mortality, morbidity and risk factors are appropriate. However, the IHME also used data related to earthquakes in Italy released by the British Broadcasting Corporation (BBC) News, which is inappropriate.

Lithuania: There are many important data sources, which are used at national level but are not used for BoD calculations by IHME. For example, morbidity data (Health Insurance Fund), national health interview survey data (Institute of Hygiene) and other data sources are presented at sveikstat.hi.lt.

Slovenia: The National Public Health Institute of Slovenia is collaborating with IHME. In the last round the mortality data sources were checked, which were the same as used for national health statistics. There are some missing data sources for risk factors (i.e., including two types of periodic surveys: 1. EHIS [European Health Interview Survey - 2nd wave] and 2. CINDI [Countrywide Integrated Non-communicable Disease Intervention-WHO]). The Drug Prescription Database is often used for morbidity estimates.

V. Discussion

The results of this study provided an overview of priority health conditions and risk factors contributing to the overall burden (i.e., DALYs) and health system performance in European countries by comparing the national health statistics with IHME calculated estimates. This comparison highlighted certain differences in ranking of priority health conditions and risk factors in some European countries. These differences may be due to additional data sources used by the individual country for national health reporting, choice of a standard population and the use of different methodological choices to calculate BoD estimates.

1. Different data sources

Some countries mentioned the additional data sources used in their routine calculations of national statistics, which are not included in the list of IHME and can influence the ranking of health conditions and risk factors produced. Low-quality data challenge to calculate BoD estimates.

2. Choice of standard population

The choice of the standard population strongly influences the disease ranking [4]. For example, in Belgium as mentioned under "Age-standardized mortality rates by top ten conditions 1990 – 2017", cardiovascular diseases are ranked as the top contributing factor to the burden of morality by using the European standard population. However, by changing the standard population from the European population to the world population, this ranking changes and cancer mortality has become the first contributing factor to the burden of mortality as used by IHME.

3. Use of different methodological choices

Several methodological challenges were identified by some European countries who produce their own BoD estimates, which limits the comparison of GBD estimates with national BoD estimates and national statistics. For example, differences in prevalence rates and duration parameters, use of different life tables to estimate YLL, difference in estimation methods used to calculate age-standardized rates, to redistribute garbage codes and invalid ICD-10 codes, use of same severity distributions across countries and regions [5], estimation of disability weights at disease level or sequelae level, comorbidity adjustment methods, etc.

Some countries mentioned that the lack of information on the method used to calculate IHME estimates makes it challenging to compare them with estimates from national studies.

4. The difference in priority health conditions and risk factors

Some countries mentioned that ranking of mortality estimates due to some health conditions do not correspond to the IHME ranking. For example, *in Romania*, mortality rates for cardiovascular diseases and cancer are under-estimated by IHME as compared to national statistics and premature mortality for cardiovascular diseases are over-estimated by IHME. *In Serbia*, mortality rates for cardiovascular and respiratory diseases were under-estimated by

IHME as compared to the Serbian BoD study in 2000. In Scotland, the mortality rates due to substance use tend to have higher numbers in national death register. *In France*, the trend of musculoskeletal disorders, mental health and neurological diseases contributing to the disability have changed since 2010, and occupational risks are underestimated.

Strengths and limitations

To our knowledge, this is the first study describing the country health profiles and comparing with national health statistics in individual countries. These results emphasize the importance of good quality data sources, choice of a standard population and methods used to calculate BoD estimates, which can influence the estimates and the ranking of health conditions and risk factors.

There are some limitations in this study. *First,* the majority of the countries did not calculate GBD estimates using their own data to compare with IHME calculated estimates. The countries only compared the IHME trends with their national health statistics of their country, which are based on prevalence and incidence estimates. *Second,* it is difficult to compare the country health profiles with national health statistics and to assess whether the differences in IHME estimates are due to the actual differences in population health or whether these are the result of different data sources, standard population and methodological choices. *Third*, the IHME estimates were not stratified by different age structures and sex to explain differences at various levels. *Fourth,* grouping of various health conditions such as neurological disorders and cancers are too general, which may not reveal the underlying the variations in estimates of individual diseases grouped under one category. As an example of cancer, the country health profile did not describe as breast cancer, lung cancer, prostate cancer, etc.

Implications for policy and research

The results of this comparison highlight the importance of key aspects such as different data sources, choice of a standard population and different methods used to calculate BoD estimates when developing BoD studies. Therefore, from perspectives of European and intra-country comparisons at local levels, the comparability and consistency of estimates across diseases, can strongly influence the local, national and European levels policy decisions. There are two possibilities: *first*, if European countries are relying on IHME estimates, it is important to share good quality and updated data sources with IHME to improving the calculation of their GBD estimates. *Second*, if European countries want to calculate their own BoD estimates, it is essential to understand the rationale of using various methodological approaches in their country contexts. As very few European countries have the capacity to calculate their own BoD estimates, therefore, to build this capacity among European countries is of prime importance.

From the perspectives of comparison of estimates among European countries, a European data infrastructure is required, which could support to establish the European BoD estimates, as a means for supporting evidence-based decision-making. This would allow sharing similar data sources and common methods used to calculate BoD estimates at EU level.

VI. Conclusions

These results highlight some variations in priority health conditions based on national health reporting or disease assessments in individual countries. Few countries have a formal, complete BoD assessment, i.e. comprehensive, updated, and repeated over time to identify trends. Even the countries who develop their own BoD study, the inter-country comparability still remains an issue due to different data sources, the choice of a standard population, and different methodological approaches. The use of GBD metrics (YLL, YLD, DALYs) is neither mandatory nor necessary when developing a BoD study. However, in the absence of a defined set of principles, countries may find it difficult to compare their estimates to their peers, and to monitor the trends of health conditions and risk factors over time.

VII. References

1. IHME: Country Health Profiles: http://www.healthdata.org/results/country-profiles.
2. Badjadj L, Chan Chee C: Hospitalization and ambulatory care for mood disorders in psychiatric settings in metropolitan france, 2010 - 2014. *BEH (Bulletin épidémiologiue hebdomadaire)* 2017.
3. Chee CC: Hospitalization and ambulatory care of patients with anxiety disorders in public psychiatric settings in metropolitan France, 2010 - 2014. *BEH (Bulletin épidémiologiue hebdomadaire)* 2018.
4. Wyper GMA, Grant I, Fletcher E, McCartney G, Fischbacher C, Stockton DL: How do world and European standard populations impact burden of disease studies? A case study of disability-adjusted life years (DALYs) in Scotland. *Archives of Public Health* 2020, 78(1):1.
5. Wyper GMA, Grant I, Fletcher E, Chalmers N, McCartney G, Stockton DL: Prioritising the development of severity distributions in burden of disease studies for countries in the European region. *Arch Public Health* 2020, 78:3.

VIII. Additional files

A. Additional file 1

Description of GBD metrics used to describe country health profiles

Figure 1 shows the observed and expected life expectancy. Expected values are based on a country's expected performance according to its Socio-demographic Index (SDI) (computed using the geometric mean of income per person, educational attainment in the population older than age 15 years, and total fertility rate).[1] Please note that GBD does not publish expected life expectancy for the European Union as a whole, which is why figures were used for Western Europe for illustrative purposes.

Figure 2 shows age-adjusted mortality rates by major conditions (top ten).

Figure 3 shows Years of Life Lost (YLLs). These are the years of life lost due to premature mortality; estimated as the product of deaths and the remaining standard life expectancy at the age of death. It is computed as how many years of life are lost due to a person dying at a particular age and did not live to the full life expectancy possible. The trends are usually very similar to mortality trends.

Figure 4 shows morbidity, as Years lived with disability (YLDs). These are years lived in less than ideal health. This includes health loss that may last for only a few days or a lifetime. YLDs are estimated from the prevalence of diseases and injuries, and the corresponding disability weight for each sequela. YLDs adjusted for disability and co-morbidity are the final YLDs. Trends in morbidity often show markedly different patterns from mortality, often 'flatlining' whereas mortality decreases.

Figure 5 shows the combined burden of mortality and morbidity, Disability-adjusted life years (DALYs). These are the years of healthy life lost due to premature death and disability. DALYs are the sum of years of life lost (YLLs) and years lived with disability (YLDs). It is the major metric used in burden of disease assessments. Its shape is usually heavily influenced by the mortality component.

Figure 6 shows the DALYs lost to all causes, distributed by most common risk factors. GBD uses a 3-category structure of risks, i.e. behavioural, metabolic and environmental, and estimates single risk factor – health outcome pairs. The amount of DALYs (their PAFs [Population Attributable Fractions]) reflects known risk factors, and therefore represents a fraction of total DALYs, as conditions such as e.g. dementia, musculoskeletal disorders or cancer are only partly explained by underlying risk factors.

Figure 7 attempts to illustrate the contribution of individual causes to changes in disease burden. The columns present year-on-year changes in rates, broken down by major causes.

---

1 http://www.healthdata.org/acting-data/new-way-measuring-development-helps-assess-health-system-performance
http://ghdx.healthdata.org/record/ihme-data/gbd-2015-socio-demographic-index-sdi-1980%E2%80%932015

This allows explaining, which causes have most contributed to changes in burden. A frequent finding across countries is the year-on-year decrease in cardiovascular disease rates.

Figure 8 presents values for the Healthcare Access and Quality Index (HAQ)[2]. This is a measure of amenable mortality based on GBD mortality rates. It is based on 32 underlying causes of death. The HAQ summarizes amenable mortality (premature deaths theoretically avoidable by access to and receipt of high quality medical care) into an index, which takes values from 0-100 where 100 is best achievable performance. Amenable mortality refers to deaths, which are thought to be avoidable in a defined set of conditions by optimal access to and quality of medical care. The higher the value of the index the more likely people are able to access high quality effective care. It has been used as a comparative measure of healthcare and health system performance for years. The box-and-whiskers plot shows the distribution of HAQ values for Member States, with the individual country highlighted as a red dot. This is the main index used by GBD to measure health system performance.

Here is an example of interpretation using the England GBD health profile

Figure 1, life expectancy

England outperforms its expected value. However, in recent years, improvements in observed LE have stalled, while the 'expected trend' is meant to be continuing to rise. Compare with other EU countries where observed life expectancy is below the expected (e.g. Hungary, Romania) or countries where life improvement has not only stalled, but has deteriorated (Scotland, Finland).

Figure 2, mortality

In England, mortality rates have greatly decreased for cardiovascular disease, and to minor extent for cancer. The recent slowing down in life expectancy rise is mirrored by the behaviour of these two conditions. This pattern is similar in most countries, in that CVD and cancer account for most of the burden of mortality and therefore largely determine life expectancy. The individual shape of curves is however different between countries, perhaps more so for cancer than for CVD.

[2] see http://www.healthdata.org/results/country-profiles/haq and http://www.healthdata.org/node/6446

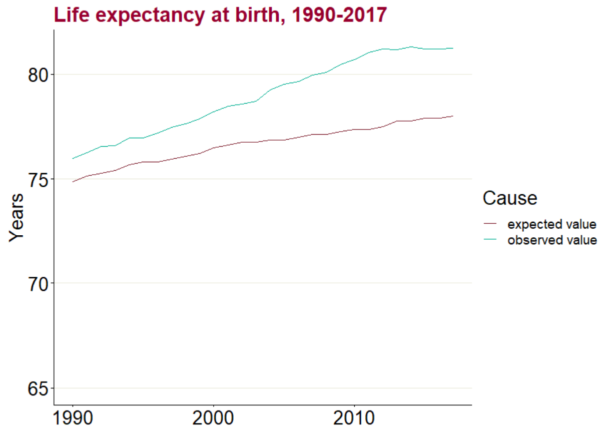

Fig 1, Life expectancy, observed vs expected

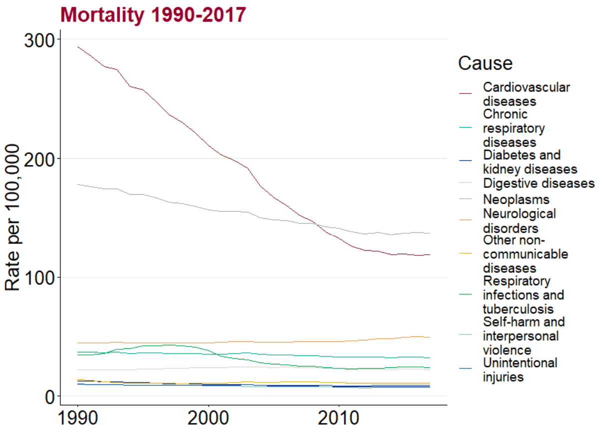

Fig. 2, mortality

Figure 3, premature mortality

The values are based on mortality data and reflect the pattern of overall improvement until the recent stalling.

Figure 4, morbidity

While mortality has massively improved, telling something about the health system's ability to protect the population from dying of a disease, morbidity has stayed mostly unchanged, in some cases even worsening, such as MSK. In fact, in countries such as England, the main challenge is morbidity, not mortality.

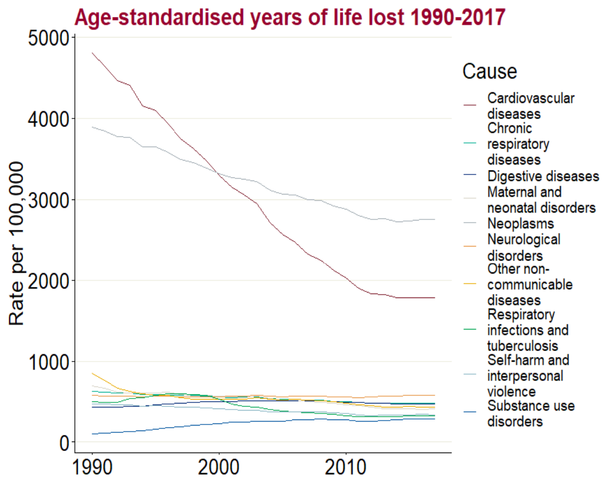

Fig. 3, premature mortality

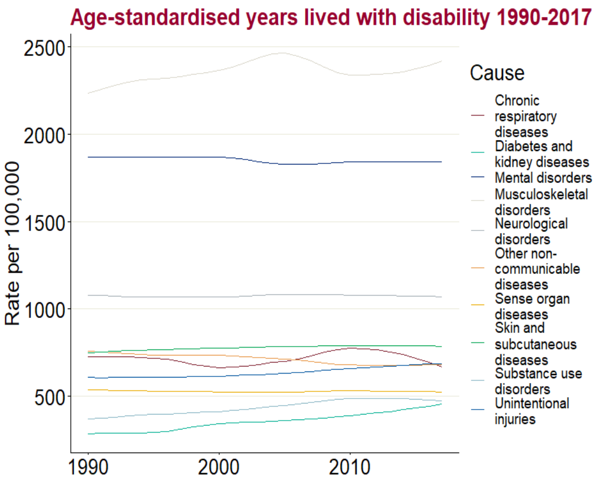

Fig. 4, morbidity

Figure 5, combined burden

DALYs are the combination of YLLs (premature mortality) and YLDs (morbidity) giving an appreciation of the overall burden. The shape of the curves is heavily influenced by premature mortality, as the rates per 100,00 of YLLs resp. YLDs allow to identify. Nevertheless, MSK and mental health figure prominently.

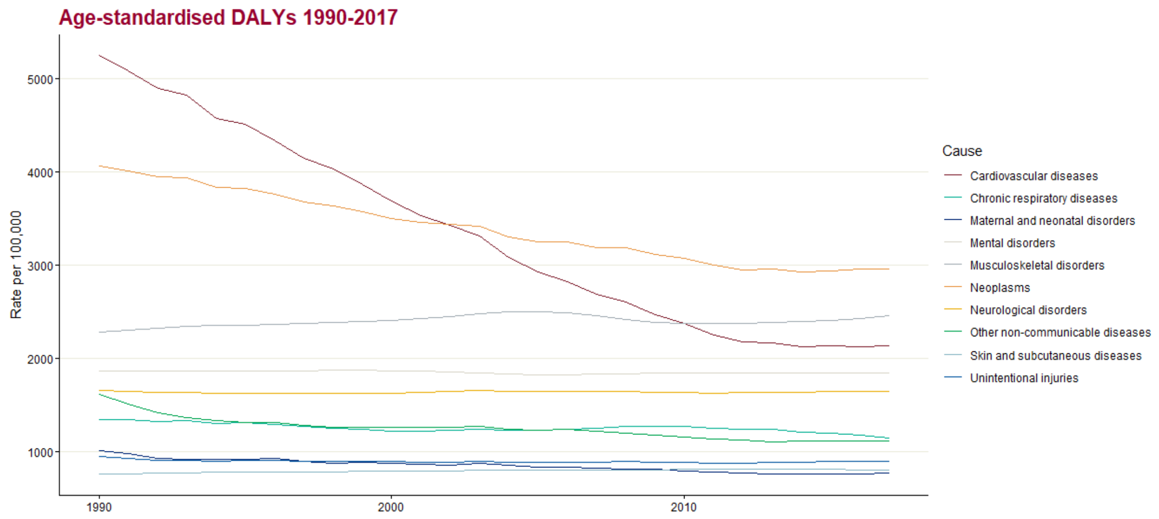

Fig. 5, combined burden

Figure 6, risk factors

This is the contribution of individual risk factors to the overall burden, measured in DALYs. Given that the question for the UK is "what has contributed to the recent stalling in life expectancy", this figure starts unravelling how risk factors have improved over the years, and how several have stopped decreasing in recent years. Risk factors like dietary risks or high blood pressure, whose contribution to the burden had been diminishing steadily over the previous years, no longer decrease in importance in recent years.

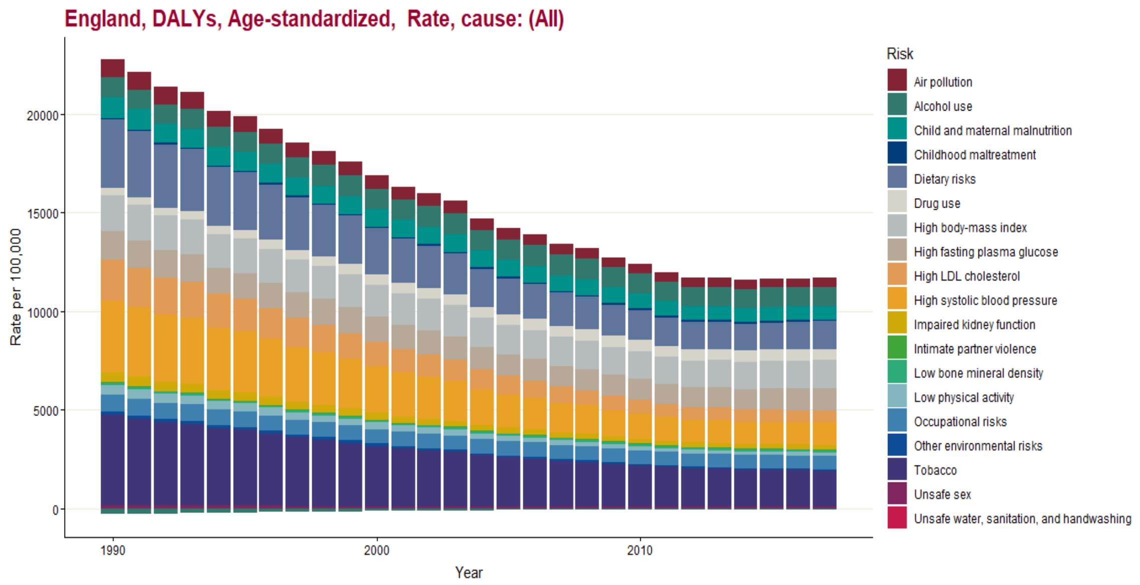

Fig. 6, risk factors

Figure 7, DALY breakdown

As mentioned above, this chart shows which year-on-year decreases in main conditions account for overall changes in the burden of disease. CVD and cancer, and to a lesser extent respiratory conditions, brought about major improvements between 1990 and roughly 2010 and stalled ever after. This pattern is found in most comparable countries.

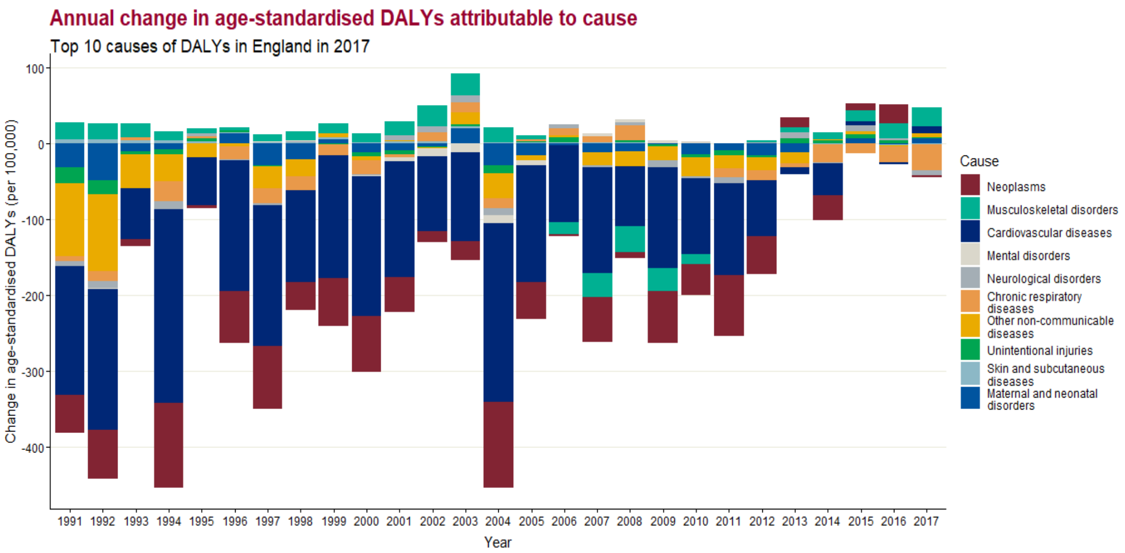

Fig. 7, DALY breakdown

Figure 8, Healthcare Access and Quality Index

This proxy measure for quality of health services shows that in England testicular cancer, rheumatic heart disease, leukaemia, and chronic kidney disease are rather well treated, reaching the highest percentile of the index. CVD and a range of cancers are equally among the higher levels of the distribution, confirming the contribution of the health services to the improvements in life expectancy, but not the recent stalling.

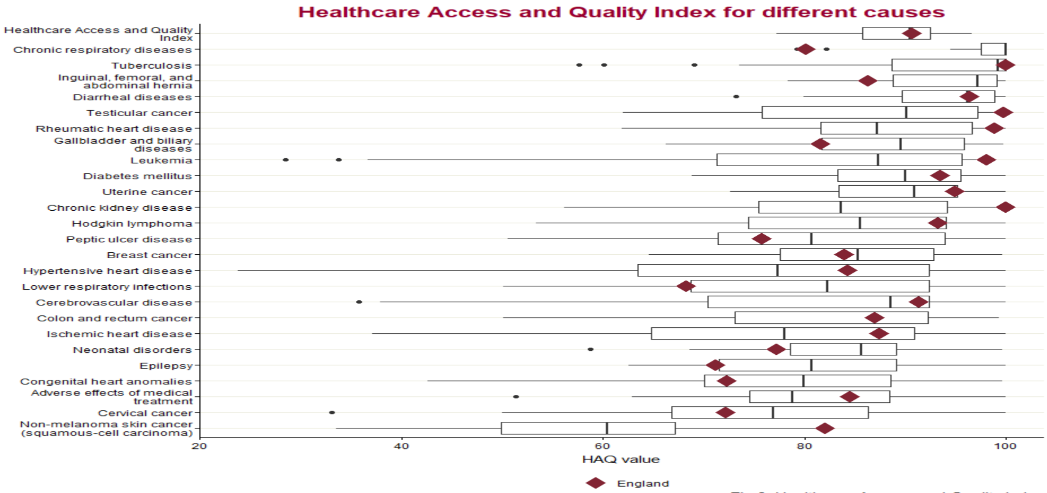

Fig.8, Healthcare Access and Quality Index

B. Additional file 2

Abbreviation of European Countries with respondents' information

S/No	Abbreviations	Country	First name	Last name	Institute
1	AT	Austria	Robert	Griebler	Austrian National Public Health Insititue (GÖG)
2	BE	Belgium	Brecht	Devleesschauwer	Sciensano, National Public Health institute
3	HR	Croatia	Jelena	Dimniakovic	National Institute of public health, division of health informatics and biostatistics
4	CY	Cyprus	Vasos	Scoutellas	Health Monitoring Unit; Ministry of Health
5	CZ	Czech Republic	Ondřej	Májek	Institute of health information and statistics of the Czech Republic
6	DK	Denmark	Janne	Tolstrup	National Institute of Public Health, Denmark
7	EE	Estonia	Jane	Idavain	National Institute for Health Development
8	ENG	England	Jürgen	Schmidt	Public Health England
9	FI	Finland	Hanna	Tolonen	Finnish Institute for Health and Welfare (THL), Helsinki
10	FR	France	Anne	Gallay	Santé Publique France
11	DE	Germany	Alexander	Rommel	Robert Koch Institute
12	EL	Greece	Spyridon	Goulas	National Organization for Health Care Services Provision [EOPYY]
13	HU	Hungary	Juhasz	Attila	Ministry of Health
14	IE	Ireland	Shoena	Gilsenan	Department of Health Service Executive, Ireland
15	IT	Italy	Brigid	Unim	Department of cardiovascular, Endocrine-metabolic Diseases and Aging
16	LV	Latvia	Laura	Isaieya	Ministry of health
17	LT	Lithuania	Ausra	Zelviene	Institute of Hygiene, Health information Centre
18	MT	Malta	Sara	Cuschieri	Centre of Molecular medicine and Biobanking, University of Malta
19	NL	Netherlands	Henk	Hilderink	RIVM, National Institute of Public Health and Environment, The Netherland
20	NO	Norway	Simon	Øverland	Norwegian Institute of Public Health
21	PL	Poland	Anna	Weszka	The Agency for Health Technology Assessment and Tariff System, Warsaw
22	PT	Portugal	Ricardo	Assunção	National Institute of Health Dr. Ricardo Jorge, Food and Nutrition Department, Lisbon
23	RO	Romania	Ciprin	Ursu	National Institute of Public Health
24	RS	Serbia	Milena	Šantrić Milićević	University of Belgrade, Faculty of Medicine, Institute of Social Medicine, School of Public Health and Health Management

25	SK	Slovakia	Jan	Cap	National Health Information Centre, Slovakia
26	SI	Slovenia	Tina	Lesnik	National Institute of Public Health [NIJZ], Ljubljana
27	ES	Spain	Rodrigo	Sarmiento Suarez	National School of Public Health, Instituto de Salud Carlos III
28	SE	Sweden	Emilie	Agardh	Karolinska Institute of Sweden
29	SC	Scotland	Ian	Grant	Public Health Scotland, Edinburgh
30	WL	Wales	Ronan	Lyons	Swansa University, Wales

C. Additional file 3

The 31 country health profiles can be accessed and downloaded through clicking following electronic link:

https://partage.santepubliquefrance.fr/public/folder/PYP5c1ltkUyhDSqwmW_88A/Country%20Health%20Profiles

This link is valid until April 1, 2021. If needed to access these country health profiles, you can contact the following address: Romana.HANEEF@santepubliquefrance.fr
